# Development and Validation of 697 Novel Polymorphic Genomic and EST-SSR Markers in the American Cranberry (*Vaccinium macrocarpon* Ait.)

**DOI:** 10.3390/molecules20022001

**Published:** 2015-01-27

**Authors:** Brandon Schlautman, Diego Fajardo, Tierney Bougie, Eric Wiesman, James Polashock, Nicholi Vorsa, Shawn Steffan, Juan Zalapa

**Affiliations:** 1 Department of Horticulture, University of Wisconsin-Madison, 1575 Linden Dr. Madison, WI 53706, USA; E-Mail: tcbougie@wisc.edu; 2 National Center for Genome Resources, 2935 Rodeo Park Dr. East, Sante Fe, NM 87505, USA; E-Mail: dfajardo@ncgr.org; 3 USDA-ARS, Vegetable Crops Research Unit, University of Wisconsin, Madison, WI 53706, USA; E-Mails: eric.wiesman@ars.usda.gov (E.W.); shawn.steffan@ars.usda.gov (S.S.); 4 USDA-ARS, Genetic Improvement of Fruits and Vegetables Laboratory, Rutgers University Chatsworth, NJ 08019, USA; E-Mail: james.polashock@ars.usda.gov; 5 Blueberry and Cranberry Research and Extension Center, Rutgers University, Chatsworth, NJ 08019, USA; E-Mail: vorsa@aesop.rutgers.edu

**Keywords:** microsatellite, SSR Mining, EST, Ericaceae

## Abstract

The American cranberry, *Vaccinium macrocarpon* Ait., is an economically important North American fruit crop that is consumed because of its unique flavor and potential health benefits. However, a lack of abundant, genome-wide molecular markers has limited the adoption of modern molecular assisted selection approaches in cranberry breeding programs. To increase the number of available markers in the species, this study identified, tested, and validated microsatellite markers from existing nuclear and transcriptome sequencing data. In total, new primers were designed, synthesized, and tested for 979 SSR loci; 697 of the markers amplified allele patterns consistent with single locus segregation in a diploid organism and were considered polymorphic. Of the 697 polymorphic loci, 507 were selected for additional genetic diversity and segregation analyses in 29 cranberry genotypes. More than 95% of the 507 loci did not display segregation distortion at the *p* < 0.05 level, and contained moderate to high levels of polymorphism with a polymorphic information content >0.25. This comprehensive collection of developed and validated microsatellite loci represents a substantial addition to the molecular tools available for geneticists, genomicists, and breeders in cranberry and *Vaccinium.*

## 1. Introduction

The American cranberry (*Vaccinium macrocarpon* Ait.) is a diploid (2n = 2x = 24), woody perennial in the family *Ericaceae* with an estimated genome size of 470 Mb [1]. Like many members of this family, it is uniquely adapted to life in a peat bog and can thrive in acidic, nutrient poor soils [2]. The *Vaccinium* genus includes other important commercial berry species, such as lingonberries, huckleberries, bilberries, and multiple species of blueberries. Together with cranberry, the fruits of these plants are increasing in popularity and consumption because of the potential health benefits provided by their array of phytochemical constituents. For example, various studies have shown that *Vaccinium* fruits contain high concentrations of anthocyanins and other polyphenolic antioxidants [3] and that cranberry fruit proanthocyanidins can help prevent urinary tract infections and various periodontal diseases [4,5,6]. 

The growing importance of cranberries and cranberry products has created a demand for new cultivars, which meet the economic, social, and environmental demands of cranberry growers, processors, and consumers. The industry currently relies on only a handful of cultivars with a narrow genetic base [7]. Cranberry improvement through classical breeding approaches has been limited but successful; however, most commercial varieties are separated from their wild brethren by only a few breeding and selection cycles [8]. Because of the increased demand for new cranberry cultivars, many recent investigations have aimed to increase the genetic resources available for cranberry molecular crop improvement. Multiple sets of microsatellite or short sequence repeat (SSR) markers have been developed in blueberry and transferred to cranberry or have been developed by mining cranberry next generation sequencing data [9,10,11]. However, despite these efforts to develop genetic resources, fewer than 200 molecular markers have been tested and validated, and only 136 markers have been added to a cranberry genetic linkage map [10]. 

Abundant genome-wide molecular markers are a prerequisite for initiating a marker-assisted selection (MAS) program. Therefore, the primary intent of this study was to increase the number of markers available for genetic research in cranberry by testing and validating polymorphic SSR loci in a set of genotypes of diverse, but known origins. The SSR loci developed herein will allow for the identification of quantitative trait loci (QTL) and candidate genes of agronomic importance. This information will be essential for the development of innovative plant breeding systems and MAS programs aimed at generating and releasing new cultivars adapted to meet the current and future challenges of the cranberry industry. Broader implications may follow by adapting these markers for use in comparative genomic and evolutionary studies within the genus *Vaccinium* and the family *Ericaceae*.

## 2. Results and Discussion

### 2.1. SSR Search and Primer Design

Continued advances in Next Generation Sequencing technologies have made genetic research and molecular marker development increasingly available and affordable to “minor” crops [12,13]. As a result, recent efforts have drastically increased the level of organellar [2], nuclear [14], and transcriptome [14] sequence data existing for *V. macrocarpon*, a species which has traditionally lacked genetic and genomic resources. Furthermore, multiple studies have been conducted characterizing the presence of SSRs within the nuclear genome and transcriptome [11,14]; however, these studies have only developed and tested a limited number of these loci. Currently, less than 200 validated nuclear SSR markers are available for cranberry [10], which equates to only one marker for every 3.4 Mb of the estimated 470 Mb nuclear genome [14]. Therefore, this study aimed to increase the number of molecular markers available for conducting comparative genomic analyses, linkage and QTL mapping, genome wide association, and diversity studies in cranberry and the *Vaccinium* genus.

**Figure 1:**
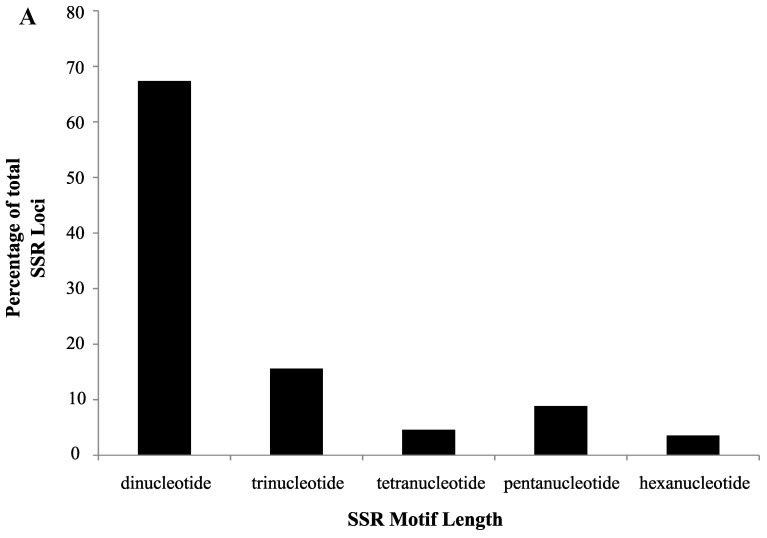

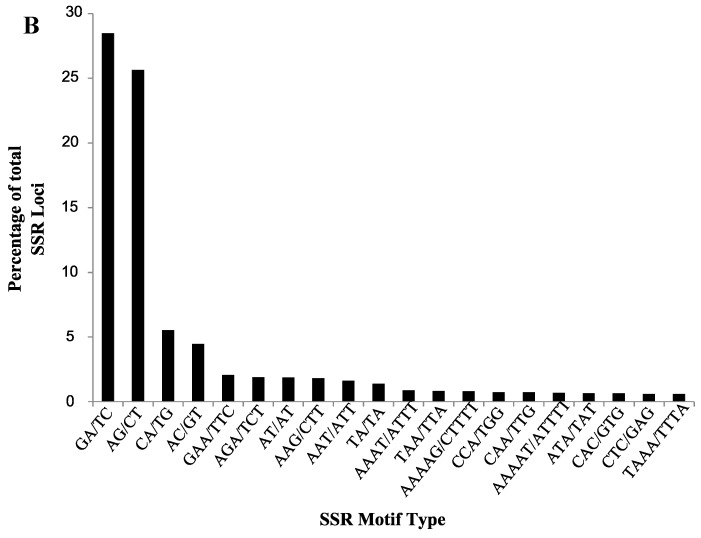
(**A**) The percentage of cranberry (*Vaccinium macrocarpon)* short sequence repeat (SSR) loci identified using SSR locator by motif length (**B**) and by motif type when the motif type represented more than 0.5% of the total detected loci.

The current SSR search detected 7557 perfect SSRs within an assembly of 237,651 *V. macrocarpon* nuclear scaffolds [15] using SSR locator [16], which was nearly 90% fewer microsatellites than either of the previous studies discovered [11,14]. However, the current investigation employed much stricter parameters (*i.e.*, a minimum of four repeat units and a minimum length of 18 bp* versus* a minimum of three repeat units and a minimum length of 12 bp) in order to increase the likelihood of identifying polymorphic loci. The frequency and distribution of SSR motif types were consistent with those found in the most recent SSR characterization [14]; the most common motif length within the nuclear scaffolds were di-nucleotides (67.4%) and the GA/TC was the most abundant motif type (28.5%) (Figure 1). Requiring tetra-nucleotides to contain a minimum of five repeat units caused them to be underrepresented in this study, only 4.6% compared to nearly 20% of the total in past analyses [11,14] (Figure 1).

The 7557 microsatellite containing nuclear scaffolds were screened for suitable flanking sites for PCR primer design using WebSat [17], and primers were successfully designed for 816 loci. An additional set of 7772 sequences containing EST-SSRs previously identified, but not developed [14] were also screened and 163 primer pairs were designed for di-nucleotide and tri-nucleotide motifs with long repeat lengths. In total, primers were designed and synthesized for 979 new SSR loci; 568 SSRs were from genomic sequences and 129 were from EST sequences.

### 2.2. Validation of Cranberry SSR Loci

Of the 979 designed and tested primer-pairs in this study, 896 (91%) amplified products in an initial polymorphism screen of four cranberry cultivars (Table 1; “Crimson Queen”, “Mullica Queen”, “Stevens”, and “Ben Lear”). A total of 697 (71%) of the 979 developed primer-pairs produced a maximum of two fragments per individual and were considered putative polymorphic loci (Table S1, Table S2 and Table S3). The remaining 243 primer-pairs which produced amplicons in the 4 cranberry cultivar either displayed monomorphic allele patterns (9%) or amplified more than two alleles which is not consistent with single locus segregation in a diploid organism (11%). Sequences containing the 697 polymorphic SSR loci validated in this study were deposited in GenBank, and the polymorphic EST-SSR loci were annotated using Blast2Go (Table S1) [18]. About 73% of the polymorphic loci contained alleles, which distinguished the cultivars Crimson Queen and Mullica Queen, and these 507 primer-pairs were used in an expanded genetic diversity analysis with 13 cranberry cultivars and a segregation analysis with 16 derived progeny from a “Crimson Queen” × “Mullica Queen” cross. 

**Table 1:** Cranberry (*Vaccinium macrocarpon)* genotypes used in the short sequence repeat (SSR) diversity and SSR segregation analyses, their source, pedigree information, and release date. Pedigree information includes either the genotype’s parents if it is a hybrid or the location where the clone was found if it is a native selection. The release date of native selections is the date the clone was found. Native Selections are indicated with an “*”.

Status	Name	Source	Pedigree	Release Date
Cultivar	Stevens	NCGR (PI 614078)	McFarlin * × Potters Favorite *	1950
Cultivar	GH1	E. Grygleski, Wisconsin	McFarlin * × Searles *	2004
Cultivar	Ben Lear *	NCGR (PI 554983)	Native Selection, WI, USA	1901
Cultivar	Crimson Queen	Rutgers University	Stevens × Ben Lear *	2006
Cultivar	Demoranville	Rutgers University	Franklin × Ben Lear *	2006
Cultivar	Franklin	NCGR (PI 554998)	Early Black * × Howes *	1950
Cultivar	Howes *	NCGR (PI 614076)	Native Selection, MA, USA	1843
Cultivar	Lemunyon *	NCGR (PI 554985)	Native Selection, NJ, USA	1960
Cultivar	Sundance	UW-Madison	Stevens × Ben Lear *	2011
Cultivar	HyRed	UW-Madison	Stevens × Ben Lear *	2003
Cultivar	LoRed	UW-Madison	Stevens × Ben Lear *	Unreleased
Cultivar	Mullica Queen	Rutgers University	(Howes * × Searles *) × LeMunyon *	2007
Cultivar	BG	E. Grygleski, Wisconsin	Beckwith × GH1	2012
Progeny	CNJ02-1-159	Rutgers University	Mullica Queen × Crimson Queen	Unreleased
Progeny	CNJ02-1-160	Rutgers University	Mullica Queen × Crimson Queen	Unreleased
Progeny	CNJ02-1-161	Rutgers University	Mullica Queen × Crimson Queen	Unreleased
Progeny	CNJ02-1-162	Rutgers University	Mullica Queen × Crimson Queen	Unreleased
Progeny	CNJ02-1-163	Rutgers University	Mullica Queen × Crimson Queen	Unreleased
Progeny	CNJ02-1-164	Rutgers University	Mullica Queen × Crimson Queen	Unreleased
Progeny	CNJ02-1-165	Rutgers University	Mullica Queen × Crimson Queen	Unreleased
Progeny	CNJ02-1-166	Rutgers University	Mullica Queen × Crimson Queen	Unreleased
Progeny	CNJ02-1-168	Rutgers University	Mullica Queen × Crimson Queen	Unreleased
Progeny	CNJ02-1-169	Rutgers University	Mullica Queen × Crimson Queen	Unreleased
Progeny	CNJ02-1-170	Rutgers University	Mullica Queen × Crimson Queen	Unreleased
Progeny	CNJ02-1-173	Rutgers University	Mullica Queen × Crimson Queen	Unreleased
Progeny	CNJ02-1-174	Rutgers University	Mullica Queen × Crimson Queen	Unreleased
Progeny	CNJ02-1-175	Rutgers University	Mullica Queen × Crimson Queen	Unreleased
Progeny	CNJ02-1-176	Rutgers University	Mullica Queen × Crimson Queen	Unreleased
Progeny	CNJ02-1-178	Rutgers University	Mullica Queen × Crimson Queen	Unreleased

In general, the lack of stuttering in tetra-, penta-, and hexa-nucleotide motifs compared to smaller motif classes makes allele sizing and genotyping more straightforward [19]; furthermore, the larger motif classes are sometimes preferred for population genetic studies because they tend to have slower mutation rates and a reduced risk for size homoplasy [20]. However in this study, while all of the primers designed for tetra-nucleotide, penta-nucleotide, and hexa-nucleotide SSRs produced amplicons, only 33% of those SSRs were polymorphic suggesting that investigations intending to validate large motif class SSRs should use more lenient parameters and test many more primer-pairs and individuals than were used in the current characterization. Conversely, more than 70% of primer-pairs from both the di-nucleotide and tri-nucleotide motif classes were found to be polymorphic. Therefore, investigations intending to validate markers for direct parentage analysis as in linkage mapping and QTL mapping where size homoplasy due to microsatellite mutation rates are a non-issue should focus their investments on testing primer-pairs designed for lower class SSR loci (*i.e.*, di-nucleotides and tri-nucleotides).

### 2.3. Genetic Diversity and Segregation Analyses

The 507 polymorphic (Table S1 and Table S2) SSRs tested on the panel of 13 cranberry cultivars yielded 2278 alleles with an average of 4.49 alleles and a range of 2 to 11 alleles (N_A_) per locus (Table 2). More than 80% of the 507 polymorphic SSRs tested were di-nucleotides, and the di-nucleotide motif class had greater average number of effective alleles (N_E_) (3.24), polymorphic information content (PIC) (0.59), observed heterozygosity (H_O_) (0.72), and expected heterozygosity (H_E_) (0.63) than the other motif classes (Table 2). As in the SSR search, GA/TC was the most common motif type tested in the genetic diversity analysis, and was responsible for 43% of the total alleles detected (Table 3).

**Table 2:** Cranberry (*Vaccinium macrocarpon*) genetic diversity statistics by motif length based on 13 cultivars genotyped using 507 polymorphic short sequence repeat (SSR) loci.

Motif Length	Number of Loci	Total Na	Average N_A_	Average N_E_	Average PIC	Average H_O_	Average H_E_
Dinucleotide	427	1983	4.64	3.24	0.59	0.72	0.63
Trinucleotide	72	272	3.78	2.87	0.51	0.67	0.58
Tetranucleotide	1	5	5.00	2.27	0.51	0.62	0.56
Pentanucleotide	7	18	2.57	1.85	0.37	0.66	0.45
Total	507	2278	4.49	3.17	0.57	0.72	0.62

Note: N_A_ = number of alleles; N_E_ = Effective number of alleles; PIC = polymorphic information content; H_O_ = observed heterozygosity; H_E_ = expected heterozygosity.

**Table 3:** Cranberry (*Vaccinium macrocarpon*) genetic diversity statistics by motif type based on 13 cultivars genotyped using 507 polymorphic short sequence repeat (SSR) loci.

Motif Type	Number of Loci	Total N_A_	Total N_E_	Average N_A_	Average N_E_	Average PIC	Average H_O_	Average H_E_
AC/GT	30	130	92.76	4.33	3.09	0.57	0.71	0.60
AG/CT	141	650	456.31	4.61	3.24	0.59	0.74	0.64
AT/AT	10	53	38.54	5.30	3.85	0.67	0.61	0.71
CA/TG	38	142	93.89	3.74	2.47	0.46	0.60	0.52
GA/TC	202	979	680.26	4.85	3.37	0.60	0.75	0.65
TA/TA	6	29	23.13	4.83	3.86	0.66	0.73	0.71
AAC/TTG	2	4	3.73	2.00	1.87	0.35	0.54	0.46
AAG/CTT	8	39	27.03	4.88	3.38	0.62	0.71	0.66
AAT/ATT	6	24	18.05	4.00	3.01	0.54	0.66	0.58
ACA/TGT	1	2	1.90	2.00	1.90	0.36	0.46	0.47
AGA/TCT	12	50	42.58	4.17	3.55	0.58	0.80	0.65
AGC/GCT	2	6	3.99	3.00	2.00	0.41	0.64	0.47
AGG/CCT	1	2	1.99	2.00	1.99	0.37	0.77	0.50
CAA/TTG	5	14	10.46	2.80	2.09	0.40	0.66	0.48
CAG/CTG	1	4	2.25	4.00	2.25	0.51	0.62	0.56
ATG/CAT	2	4	3.95	2.00	1.98	0.37	0.85	0.49
CCA/TGG	3	8	5.52	2.67	1.84	0.36	0.43	0.42
CTA/TAG	1	2	2.00	2.00	2.00	0.38	1.00	0.50
GAA/TTC	9	41	30.30	4.56	3.37	0.55	0.57	0.60
CTC/GAG	1	3	2.77	3.00	2.77	0.57	0.92	0.64
ATC/GAT	2	6	5.00	3.00	2.50	0.51	0.85	0.60
GCA/TGC	3	9	6.24	3.00	2.08	0.43	0.54	0.51
GTA/TAC	2	10	8.20	5.00	4.10	0.72	0.85	0.76
CAC/GTG	1	2	1.74	2.00	1.74	0.34	0.31	0.43
TCA/TGA	2	6	4.35	3.00	2.17	0.46	0.65	0.54
GGA/TCC	3	6	5.54	2.00	1.85	0.35	0.53	0.45
TCG/CGA	1	3	1.89	3.00	1.89	0.42	0.62	0.47
TAA/TTA	4	27	17.41	6.75	4.35	0.65	0.65	0.74
TATG/CATA	1	5	2.27	5.00	2.27	0.51	0.62	0.56
AAAAT/ATTTTT	1	3	1.75	3.00	1.75	0.39	0.54	0.43
AAACA/TGTTT	1	3	1.48	3.00	1.48	0.29	0.38	0.32
AAACT/AGTTT	1	2	1.95	2.00	1.95	0.37	0.69	0.49
CACCT/AGGTG	1	3	1.70	3.00	1.70	0.35	0.38	0.41
GTTTG/CAAAC	1	2	2.00	2.00	2.00	0.38	1.00	0.50
TTGGT/ACCAA	1	3	2.05	3.00	2.05	0.46	0.62	0.51
TTTTA/TAAAA	1	2	2.00	2.00	2.00	0.38	1.00	0.50

Note: N_A_ = number of alleles; N_E_ = Effective number of alleles; PIC = polymorphic information content; H_O_ = observed heterozygosity; H_E_ = expected heterozygosity.

Nearly 66% of the markers in the genetic diversity study contain high levels of genetic information according to the suggested criterion of high (PIC > 0.5), moderate (0.25 < PIC < 0.5), and low (PIC < 0.25) [21]. Of the remaining markers, 31% contain moderate and only 3% contain low levels of genetic information. The large number of alleles possible for each SSR locus allows a single SSR with *k* alleles to achieve the same genetic information content as (*k*-1) biallelic markers, such as single nucleotide polymorphisms (SNPs) [22], and therefore, it can be inferred that these 507 markers may contain an equivalent level of genetic information as more than 1000 SNPs. The 507 polymorphic markers analyzed herein are likely to have broad utility and applicability in various types of *Vaccinium* genetic studies because the relatively even distribution of SSRs throughout nuclear genomes makes them particularly useful for conducting linkage mapping, comparative genomic, and QTL mapping studies [23]. Segregation analyses using chi-square tests revealed that only 4% of the 507 markers tested on the 16 “Crimson Queen” × “Mullica Queen” derived progeny displayed segregation distortion at the *p* < 0.05 level (Table S1 and Table S2). The remaining 487 SSR markers are immediately available for resolving the current *V. macrocarpon* linkage map from 13 linkage groups to the true 12 groups and increasing the number of mapped distinct loci from only 136 markers to a number more appropriate for QTL studies [10]. The current marker set (up to 697 SSRs) combined with the previous 136 mapped markers could increase the marker density in the cranberry linkage map from an average of one marker every 3.4 Mb to one marker every 0.6 Mb.

**Figure 2:**
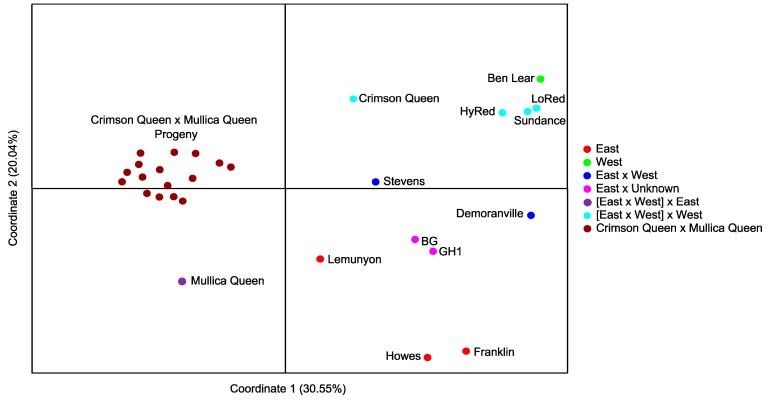
Principle Coordinate Analysis (PCoA) based on 507 microsatellite markers tested and validated on a panel of 13 cranberry cultivars and 16 Crimson Queen × Mullica Queen Progeny. Genotypes are color-coded based on the similarity of the geographic origins of their pedigrees (*i.e.*, geographic origins are specified as either east, west, or a combination of east and west of the Appalachian Mountains due to artificial selection).

The 507 SSR loci were more than sufficient to clearly separate all 29 cranberry genotypes tested in a principle coordinate analysis (PCoA) (Figure 2). The first principle coordinate accounted for 30.55% of the total genetic variation and the second principle coordinate accounted for 20.04%. Although the sample size is small, the analysis demonstrates the utility of these markers for inferring genetic and geographic relationships within cranberry. The second principal coordinate axis separated genotypes based on their geographic origins. All selections east of the Appalachian Mountains and second-generation backcrosses to an eastern parent fell below the x axis, and a wild selection from the west and the second generation backcrosses to a western parent fell above the x-axis (Table 1, Figure 2). These markers were also useful for tracking hybrid backgrounds. The first generation hybrids “Stevens” and “Demoranville”, which lay near the x-axis each have pedigrees involving one eastern and one western parent; the “Ben Lear” × “Stevens” offspring fall in a cloud between the two parents in the first quadrant; and the “Crimson Queen × Mullica Queen” (50% East/50% West) derived progeny lay in a cloud between the parents in the second and third quadrants (Table 1, Figure 2). Additional PCoA analyses which included only the 13 cultivars in the genetic diversity panel (Figure S1.A), and a random set of only 50 SSR markers rather than the 507 (Figure S1.B) displayed similar trends and utility for inferring genetic and geographic relationships within cranberry. Expansion of this genetic diversity panel in future studies to include all available cultivars and elite parents from cranberry breeding programs should resolve the parentage composition of several unknown hybrid cultivars, and expanding the panel to include wild selections from across the current *V. macrocarpon* native range will provide insights into cranberry evolutionary history and identify pockets of unsampled genetic diversity for application in future cranberry breeding systems.

## 3. Experimental Section

### 3.1. Plant Material

Commercial cranberry production relies on a small number of true-to-type clonal cultivars. Some commercial cultivars are actually clones of native wild selections previously found in North American peat bogs, and the remaining commercial cultivars are either first- or second-generation artificial hybrids of those native wild selections. In this study, a panel of 13 unique commercial cultivars (Table 1) was selected for an analysis of SSR allele diversity and polymorphism screening. In order to perform a simple test of the hypothesis that the Appalachian Mountains may serve as a genetic barrier between extant North American wild cranberries, cultivars with genetic origins from both east and west of the Appalachian Mountains were included. An additional set of 16 progeny from a “Crimson Queen” × “Mullica Queen” cross was collected for an analysis of SSR allele segregation. The germplasm for this study was provided by four different sources including: the National Clonal Germplasm Repository in Corvallis, OR; cranberry breeder Edward Grygleski; the Rutgers University cranberry breeding program; and the University of Wisconsin-Madison cranberry breeding program. Genomic DNA was extracted from lyophilized leaf tissue from individual reproductive stems of each genotype using a Macherey-Nagel (MN) Plant II kit (Düren, Germany) following the manufacturer’s instructions. 

### 3.2. Detection of Genomic and EST-SSR Markers

SSR markers were mined from a set of nuclear scaffolds greater than 100 bp that were previously reported [15]. Prior to the SSR search, all sequences were mapped against the *V. macrocarpon* plastid and mitochondrial genomes to remove organellar sequences [2,15]. *In silico* PCR was performed using primers sets for all previously published SSR markers in Geneious 6.1 [24] to locate and remove all scaffolds containing SSRs which had been previously identified and developed [9,10,11,25,26].

The remaining 237,651 remaining scaffolds were subjected to an SSR search using SSR locator with parameters set to identify motifs with repeat lengths di ≥ 9, tri ≥ 6, tetra ≥ 5, and penta through hexa ≥ 4 [16], which resulted in7557 microsatellite containing scaffolds. WebSat [17] was used to design primer pairs for all SSR loci identified above and from an additional set of 7772 EST-SSRs previously identified, but not developed or validated [14]. WebSat was used for primer design because it allows the user to visually inspect each loci to ensure that only one SSR primer pair was designed per scaffold; to redesign or discard all primer pairs containing a mononucleotide repeat of 5 or more; and to prevent possible fragment size homoplasy before primer synthesis by discarding all primer pairs with predicted PCR products containing a mononucleotide repeat of 6 or more. Major parameters selected for primer design included primer length of 19 to 25 bp (optimum 22 bp), PCR product length varying between 120 and 325 bp, GC content between 40% and 80%, and an optimum melting temperature of 55 °C. All polymorphic EST-SSRs were annotated using Blast2Go [18].

### 3.3. PCR Amplification and Fragment Analysis

Prior to synthesis, all SSR forward primers were appended with the M13 sequence (5'-CACGTTGTAAAACGAC-3') to allow for indirect fluorescent labeling of PCR products [27]. The PIG sequence (5'-GTTTCTT-3') was appended to the reverse primers to promote uniform non-templated “A” addition and to facilitate downstream genotyping [28]. PCR reactions were performed in 8 uL total volume using 3.5 µL 1× JumpStart REDTaq ReadyMix (Sigma, St. Louis, MO, USA), 1.0 µL of 15 ng/µL DNA, 2.0 µL of ddH_2_O, 0.5 µL of 5 µM forward primer, 0.5 µL of 50 µM reverse primer, and 0.5 µL of 0.5 µM M13-FAM, M13-HEX, or M13-NED primer. Thermocycling conditions included a 3 min melting step of 94 °C, followed by 33 cycles of 94 °C for 15 s, 55 °C for 90 s, and 72 °C for 2 min, and a final extension step of 72 °C for 30 min. One microliter each of FAM, HEX, and NED labeled PCR product was mixed with 10 uL formamide and a carboxy-X-rhodamin (ROX) ladder, and the pool-plexed mix was sent to the University of Wisconsin Biotechnology Center DNA Sequencing Facility for fragment analysis using a ABI 3730 fluorescent sequencer (Pop-6 and a 50 cm capillary array; Applied Biosystems, Foster City, CA, USA). Allele genotyping was performed using the GeneMarker software v 1.91 (SoftGenetics LLC, State College, PA, USA). 

### 3.4. Validation of SSR Polymorphism and Genetic Diversity Analysis

An initial polymorphism screen of all designed primers was performed using four cranberry cultivars (“Crimson Queen”, “Mullica Queen”, “Stevens”, and “Ben Lear”). The 507 primers that amplified allele patterns consistent with single locus segregation and that displayed polymorphism between Crimson Queen and Mullica Queen were used in a subsequent genetic diversity screen which included the remaining 9 cultivars in the study and in a segregation analysis using the 16 experimental hybrids (Table 1). 

The observed number of alleles (N_A_), expected number of alleles (N_E_), observed heterozygosity (H_O_), and expected heterozygosity (H_e_) were calculated for each polymorphic locus in GenAlEx 6.4 [29]. In addition, the polymorphic information content (PIC) for each locus was calculated using Cervus 3.0 [30]. Chi-Square tests were used to analyze allele segregation of the 507 loci in a panel of 16 hybrid progenies (Table 1). Finally, principal coordinate analysis (PCoA) was performed with all polymorphic loci and all 29 individuals based on pairwise genetic similarity distances, which are equivalent to Euclidean distances, as estimated by the Eigen procedure of GenAlEx 6.4 (29).

## 4. Conclusions

An extensive number of microsatellite primers located in transcribed and genomic regions were validated for *V. macrocarpon*. The 697 polymorphic loci identified, 507, which were included in genetic diversity and segregation analyses, constitute the most comprehensive set of molecular markers developed in cranberry to date. The collection of microsatellites presented herein is a substantial addition to the various molecular tools previously available. They should have immediate impacts in cranberry breeding programs, which use these SSRs and their genomic locations to identify QTL and the genetic architecture of various agronomic traits. However the long term impact of this research may be much broader by promoting studies of population genetic structure and comparative genomics within and outside cranberry aimed at increasing knowledge about the evolution and adaptation of unique characteristics in the *Vaccinium* genus and the *Ericaceae* family.

## Supplementary Materials

Supplementary materials can be accessed at: http://www.mdpi.com/1420-3049/20/02/2001/s1.
