# Supplementary material for: Development and Validation of 697 Novel Polymorphic Genomic and EST-SSR Markers in the American Cranberry (Vaccinium macrocarpon Ait.)
Source: Molecules. 2015 Jan 27;20(2):2001–13. doi: 10.3390/molecules20022001 (PMC6272188; doi:10.3390/molecules20022001)
Supplement: Supplementary file 1 [file molecules-20-02001-s001.pdf]

Supplementary Materials

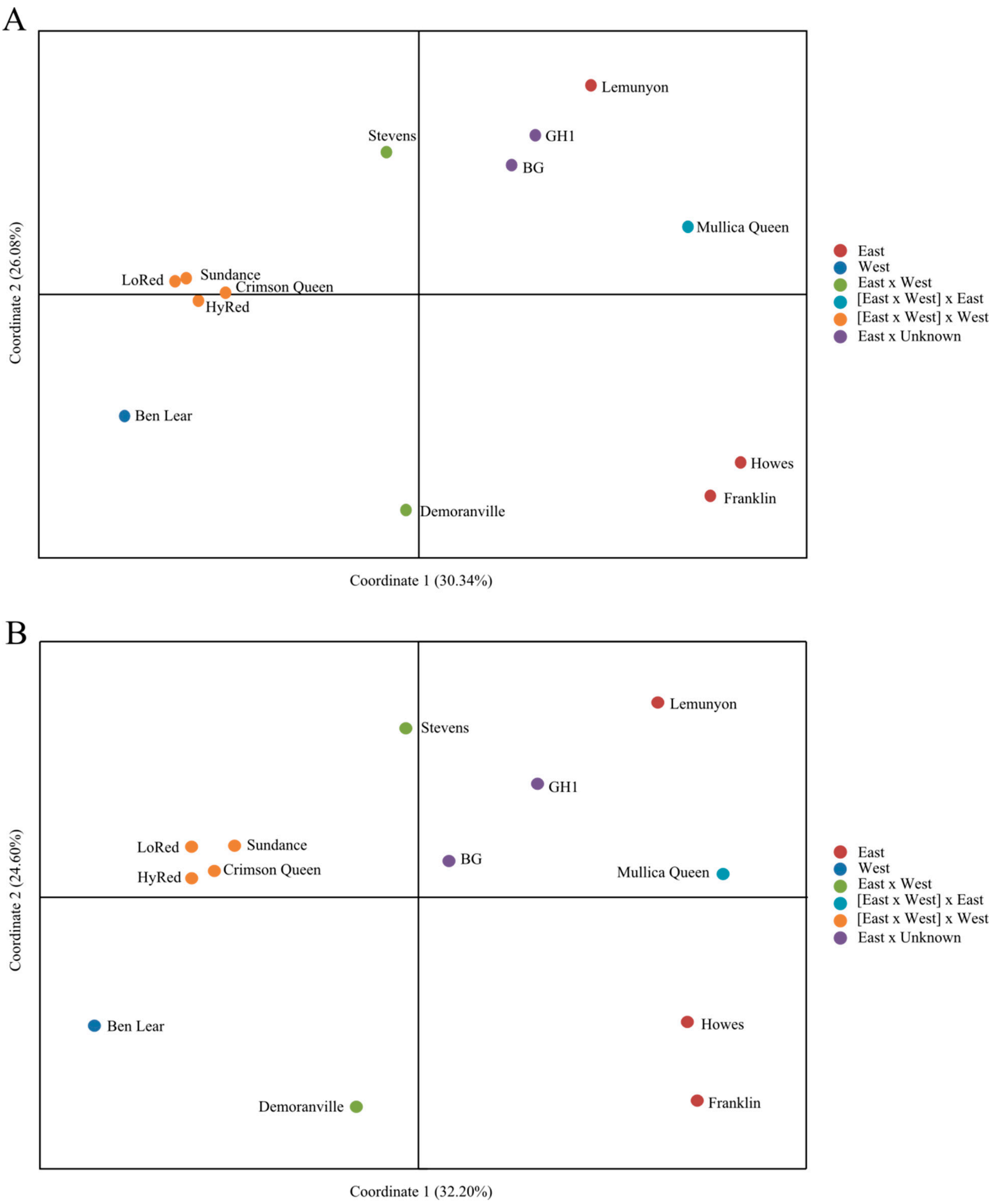

Figure S1. (**A**) Principle Coordinate Analysis (PCoA) based on 507 microsatellite markers and (**B**) 50 microsatellite markers tested and validated on a panel of 13 cranberry cultivars. Genotypes are color-coded based on the similarity of the geographic origins of their pedigrees (*i.e.*, geographic origins are specified as either east, west, or a combination of east and west of the Appalachian Mountains due to artificial selection.

Supplementary Table 1. Primer characteristics, diversity statistics, and segregation analysis statistics of 95 polymorphic EST-SSRs tested on a diversity panel of 13 cranberry cultivars and 16 progeny from a ‘Crimson Queen’ × ‘Mullica Queen’ cross.

Primer ID	Motif	Primer Sequences (5′-3′)	TM (°C)	Allele Range	Description of Putative Function	GenBank Accession Number	Heterozygosity				
							N_A	N_E	H_O	H_E	PIC
**121633_K63**	(CT)18	F:GCAGCTCTCTGTAAATTCCTT	F:55.53	**288–333**	hypothetical protein POPTR_0004s16460g	KP279163	6	4.76	1	0.79	0.76
		R:ATGGTTGAAGATGTTGATGG	R:55.77								
**128239_K63**	(GA)19	F:AAATAACGATGGCTACATCC	F:54.29	**185–207**	dna-directed rna polymerases iv and v subunit 3-like	KP279164	6	3.52	0.85	0.72	0.67
		R:GTTTGTTGATGACAATCCTG	R:53.81								
**162108_K70**	(GA)15	F:GAAGTCGAAACCCTAGCAG	F:55.1	**285–318**		KP279200	7	4.57	0.92	0.78	0.75
		R:GTCCCTCTCAGTCTCTCACTC	R:55.49								
**16720_K63**	(TC)17	F:CTACCTTTCCCTCTCCTTGT	F:55.05	**164–185**	cellulose synthase-like protein e1-like	KP279159	6	4.51	0.85	0.78	0.74
		R:AGTTGAAGCTGAGAATTGTACC	R:55.31								
**172672_K70**	(GA)16	F:GATAGTTGTATGCGCTGTAAGA	F:54.97	**325–356**		KP279201	8	5.83	1	0.83	0.81
		R:GTTACCCGAATGAACAGGT	R:54.87								
**187382_K70**	(TC)16	F:CCTCCATTCTCTCTCCTACTAA	F:54.52	**240–252**	probable receptor protein kinase tmk1-like	KP279202	7	5.83	1	0.83	0.81
		R:CGTTTCTTTTCTTCTCTCTCTC	R:54.43								
**198358_K70**	(AG)14	F:AATCGTCTGTTGCTCAATGT	F:55.74	**313–362**		KP279203	5	3.05	0.62	0.67	0.62
		R:AACCATACTTACCACAACCAGT	R:55.23								
**1trimcontig175770**	(CT)16	F:GTGTAGCTTGGAAAATAGGAGT	F:54.48	**327–346**	tonoplast monosaccharide transporter2 isoform 1	KP279220	6	4.83	1	0.79	0.76
		R:ACTAGGGAGCGAGAGAGAGTA	R:55.13								
**1trimcontig176042**	(AGA)13	F:CCGTTGTTGTTCTTCTGTAGT	F:54.55	**226–234**		KP279221	3	2.18	0.62	0.54	0.46
		R:TTCAACCTCTGAAGCCTCT	R:55.04								
**1trimcontig176303**	(AG)14	F:GCTGATTAGGTTCACTTTCTTC	F:54.97	**190–196**	shock protein src2	KP279222	4	2.99	0.85	0.67	0.6
		R:TTTCTTCACCTCTTTCTCTCTC	R:54.64								
**1trimcontig176861**	(TC)16	F:ATGGATGTATCTTGACAGGC	F:54.97	**131–183**		KP279223	8	6.26	1	0.84	0.82
		R:CTGCTGTTCATTTCTCTGTG	R:54.42								
**1trimcontig178358 ***	(GA)15	F:AATTGAACGATCCCTATTCC	F:55.58	**224–249**		KP279224	7	4.69	0.77	0.79	0.76
		R:GATTCATCACCCCTTGAAC	R:55.18								
**1trimcontig179737**	(CT)16	F:CCTCCAACTTCTTCATCTTCT	F:55.18	**204–220**	PREDICTED: uncharacterized protein LOC102600962	KP279225	5	4.76	1	0.79	0.76
		R:ACTGGTAACTCCTCAGAAACAG	R:55.23								

Table S1. *Cont.*

Primer ID	Motif	Primer Sequences (5′-3′)	TM (°C)	Allele Range	Description of Putative Function	GenBank Accession Number	Heterozygosity				
							N_A	N_E	H_O	H_E	PIC
**1trimcontig182430 ***	(AG)14	F:GAAGATGGACCTGAGTAAGAAA<br>R:CTACCATTGTGTTCTCAAACTG	F:55.21<br>R:55	**184–194**		KP279226	4	2.62	0.69	0.62	0.56
**1trimcontig209220**	(TC)16	F:GTATTTGTTCACACTCACCAGA<br>R:ACAGTTGTCGAAGCCTCAT	F:55.23<br>R:55.74	**387–421**		KP279228	7	4.36	0.92	0.77	0.74
**1trimcontig217158**	(GAA)14	F:GGAGTCGGTAAAATCAAGAA<br>R:CCAAATTCAGTAGGAGTACACA	F:54.44<br>R:54.53	**154–169**		KP279229	4	3.22	0.46	0.69	0.64
**1trimcontig237406**	(GA)15	F:TCTTAGGAAAGACGAGAACATC<br>R:GAAAGGAAGGTATGCTACAGTT	F:55.36<br>R:54.48	**314–342**		KP279230	6	3.6	0.67	0.72	0.69
**1trimcontig238080**	(AAG)13	F:AGGGGTAATCTTCACACACTTA<br>R:ACAGGCTCTTCTAATCGTTTC	F:55.44<br>R:55.37	**403–423**		KP279231	5	4.57	0.69	0.78	0.75
**1trimcontig238343**	(TG)15	F:GGTAATAGCTTTGTGATCTTGC<br>R:GATGGTGAATAAATTGCGAC	F:55.81<br>R:55.58	**291–329**		KP279232	6	4.07	0.85	0.75	0.72
**1trimcontig238795**	(AG)14	F:AGAGGGAGAGAAGAGTATGGTC<br>R:CCGTCAAGATTTGTGAAGAT	F:55.7<br>R:55.2	**262–287**		KP279233	4	2.79	0.85	0.64	0.58
**1trimcontig239742 ***	(GA)19	F:AACAAGAACAACTAAGACCACC<br>R:TACAAGTTTCAATCAGCCCT	F:55.08<br>R:54.96	**270–290**		KP279234	7	4.39	0.85	0.77	0.74
**1trimcontig326802**	(AG)14	F:TTTTCAGAGCAAGAGGAAAG<br>R:CTGTCTGTATCATGGAACTCAT	F:54.97<br>R:54.21	**201–221**	casp-like protein rcom_0464280-like	KP279235	7	5.93	1	0.83	0.81
**1trimcontig328266**	(GA)15	F:ACAGATCAAGCGAACACTAAAC<br>R:CCTGCTCCTGTTATACTACCAA	F:56.21<br>R:56.17	**235–274**	pto-interacting protein 1	KP279236	6	4.17	0.85	0.76	0.73
**1trimcontig332949**	(AG)15	F:ACCCAAACACAAAAGAACAG<br>R:GACTGCAAGTGTCTAAATGCT	F:55.2<br>R:54.78	**306–349**		KP279237	7	5.28	0.85	0.81	0.78
**1trimcontig332960**	(GA)14	F:GTCAACAGATTCAACACAACAC<br>R:CCTGCTTCTCTCTAATGAAGTC	F:55.56<br>R:55.12	**242–262**		KP279238	5	2.78	0.73	0.64	0.6
**1trimcontig337780**	(TC)14	F:CTTGATCTTGTCGCTGTAGAC<br>R:TTCCTTATCGAAATCACGAG	F:55.2<br>R:55.04	**348–364**	udp-d-apiose udp-d-xylose synthase 2	KP279239	6	5.45	1	0.82	0.79

Table S1. *Cont.*

Primer ID	Motif	Primer Sequences (5′-3′)	TM (°C)	Allele Range	Description of Putative Function	GenBank Accession Number	Heterozygosity				
							N_A	N_E	H_O	H_E	PIC
**1trimcontig339726**	(GA)17	F:TACTCATGTCGAAGCAATAGAG<br>R:CTTTAGCAGAGGAGAAACAAGT	F:54.92<br>R:54.82	**191–199**	low quality protein: uncharacterized loc101228446	KP279240	5	2.14	0.54	0.53	0.5
**1trimcontig344502**	(AG)16	F:TGGAAATGGAAAAGTCTCTG<br>R:CACCGTCTACAGTTTAAGAACA	F:55.35<br>R:54.77	**145–165**		KP279241	4	2.91	0.67	0.66	0.6
**1trimcontig351427**	(AG)17	F:GACGGCTAAATTGTAACTAACG<br>R:AGGGTCCTATCCTATCCTCTAA	F:55.91<br>R:55.22	**220–245**		KP279242	7	5.28	0.92	0.81	0.79
**1trimcontig352078**	(TC)14	F:CGTGTTTCTGTTAGATAGCTTG<br>R:CTTGTACGTGAAGATGCAAA	F:54.63<br>R:54.95	**223–229**		KP279243	4	3.65	1	0.73	0.68
**1trimcontig435620**	(GA)17	F:CAACCAGCCTTACAGTGAATA<br>R:GTCCGTTCAATTTCTTTTCC	F:55.52<br>R:56.26	**235–263**	dual specificity protein phosphatase dsp8	KP279244	8	5.54	0.92	0.82	0.8
**1trimcontig436904**	(AAT)16	F:TACCAACCACATCACACATC<br>R:CTTATGACGATCCCAGTAGC	F:54.95<br>R:54.91	**237–255**	thaumatin-like protein	KP279245	5	3.63	0.85	0.72	0.69
**1trimcontig439466**	(TC)16	F:CGAGTGGATAGTGATGATATTG<br>R:ACCAAGAGGAACTACAGGTAAA	F:54.81<br>R:54.79	**278–341**		KP279246	8	3.35	0.77	0.7	0.66
**1trimcontig439861**	(TC)15	F:CTCCTCTCTCGAATGACACTAC<br>R:TTCTTGTTGGCTGGAGATTA	F:55.72<br>R:55.97	**246–272**		KP279247	5	2.84	0.77	0.65	0.61
**1trimcontig440008**	(AG)15	F:GCAACAGGGACAGATATTTT<br>R:TACGGACTCATAGAAGGTTAGG	F:54.3<br>R:55.3	**179–195**		KP279248	6	3.38	0.69	0.7	0.66
**1trimcontig440230**	(AGA)12	F:ACACTTTGTAGGTGGTGGTTAT<br>R:ATTAGCAGTAGTCCAATCGGT	F:55.23<br>R:55.53	**235–243**		KP279249	4	3.56	0.77	0.72	0.67
**1trimcontig440337 ***	(TC)15	F:CTTGGAGTTAGCCTTTTAGTCA<br>R:CTGGAAGAGTGAAGATGGAATA	F:55.63<br>R:56.05	**153–182**	alpha-galactosidase-like	KP279250	6	4.23	0.85	0.76	0.73
**1trimcontig443603**	(GA)15	F:TGCACCTCCTCTCTCTCTAA<br>R:GGTTATGATGGTGGGAAAG	F:55.24<br>R:55.24	**147–151**	probable inactive receptor kinase at1g48480	KP279251	3	2.21	0.62	0.55	0.49
**1trimcontig444344**	(TC)18	F:CTGCTAATGTTGTTTGTTGC<br>R:TATTATCTCCCACCTAATGAGC	F:54.52<br>R:55.15	**253–270**		KP279252	6	4.83	1	0.79	0.76

Table S1. *Cont.*

Primer ID	Motif	Primer Sequences (5′-3′)	TM (°C)	Allele Range	Description of Putative Function	GenBank Accession Number	Heterozygosity				
							N_A	N_E	H_O	H_E	PIC
**1trimcontig450309**	(TCC)11	F:AAAATCAGAGGGAAGAAAGC<br>R:TATTAGCCAGTCCTCCTTTGTA	F:55.25<br>R:55.83	**144–147**	ethylene-responsive transcription factor	KP279253	2	1.99	0.58	0.5	0.37
**214102_K63**	(TG)15	F:GGTAATAGCTTTGTGATCTTGC<br>R:GATGGTGAATAAATTGCGAC	F:55.81<br>R:55.48	**219–256**		KP279165	5	3.63	0.69	0.72	0.69
**242569_K70**	(AG)15	F:GATATGAGAGACGAGGAATCAC<br>R:GTCAGTGGACGGTTTTAAGAT	F:55.39<br>R:55.82	**298–308**	octicosapeptide phox bem1p family protein isoform 1	KP279204	6	3.67	1	0.73	0.68
**251788_K63**	(AG)18	F:GATCTTTACCACTCCCCACT<br>R:GGATTCTCTGTCCATTGTTG	F:55.57<br>R:55.46	**190–208**	o-fucosyltransferase family protein isoform 1	KP279166	6	4.69	0.92	0.79	0.76
**252600_K70**	(AG)14	F:CTAGTTTAGAGTCGTCCCAAAT<br>R:AAGCACCTGAAGATAGTAGGAA	F:54.5<br>R:55.01	**204–212**	tetratricopeptide repeat protein 38	KP279205	4	2.23	0.75	0.55	0.51
**260167_K70 ***	(TC)15	F:TCAACATCTTTGGGACTTCT<br>R:GCTTGCCTAATATACTTCCAAC	F:54.73<br>R:54.99	**274–287**		KP279206	5	3.45	0.77	0.71	0.66
**281884_K70**	(AG)17	F:TCCACTATCTTTAGAATCCCAC<br>R:AGAGGATGGAGTTCCTTGATA	F:55.02<br>R:55.39	**330–357**	probable u6 snrna-associated sm-like protein lsm1	KP279207	6	3.71	0.85	0.73	0.69
**29080_K63**	(TC)14	F:ATGAAAACAGGGTAAACTGG<br>R:TCTCAACTCATAGAACTACGGA	F:54.24<br>R:54.31	**384–399**	probable gpi-anchored adhesin-like protein pga55 isoform x1	KP279160	6	3.45	0.92	0.71	0.66
**297265_K63**	(CT)14	F:GATCGTCATAACTAAGCTGGAT<br>R:GTCTCGAATCACAACAGGATA	F:55.2<br>R:55.22	**331–343**		KP279167	5	1.98	0.54	0.49	0.47
**300409_K63**	(CT)16	F:GGGGAATAGCAGGTAGTGAT<br>R:TATTTATCCACCCACTTCACAG	F:55.69<br>R:56.67	**218–235**		KP279168	5	4.39	0.77	0.77	0.74
**305731_K63**	(CT)16	F:GATTTCTTCGTGTTTCTCTCTC<br>R:TGCCTTTCTCTACTCTCTCTTC	F:54.91<br>R:54.84	**379–391**		KP279169	7	3.93	0.85	0.75	0.72
**307018_K70**	(AG)20	F:TAAAACCTTACCTCCTCTTCTG<br>R:TAACCTCGGATCTCCTTATCTA	F:54.63<br>R:54.89	**217–232**		KP279208	6	3.6	0.92	0.72	0.69
**308539_K70**	(GA)14	F:CTAAATTCTCAACATCTCTGGC<br>R:CCAAGAAGCATAAGGGATAGT	F:55.79<br>R:55.2	**304–313**	e3 ubiquitin-protein ligase sinat3-like	KP279209	4	3.56	1	0.72	0.67

Table S1. *Cont.*

Primer ID	Motif	Primer Sequences (5′-3′)	TM (°C)	Allele Range	Description of Putative Function	GenBank Accession Number	Heterozygosity				
							N_A	N_E	H_O	H_E	PIC
**308812_K70**	(TC)15	F:GAAAGGAAGGTATGCTACAGTT	F:54.48	**316–344**		KP279210	7	5.28	0.92	0.81	0.79
		R:TCTTAGGAAAGACGAGAACATC	R:55.36								
**308839_K70**	(TC)16	F:ATAATGTGTCCAGTCCCTTTC	F:55.58	**292–313**		KP279211	5	3.48	0.85	0.71	0.66
		R:TTCCTTCCTCAATCCACTC	R:55.05								
**309084_K70**	(AG)16	F:CTTCTTTTCCTCTCCACTGATA	F:55.37	**389–400**		KP279212	5	2.91	0.54	0.66	0.61
		R:CTCTCCGTTGTCCATTTCT	R:55.18								
**309124_K70**	(GA)15	F:AAAGGTCGTTAAGGCTATCAG	F:55.42	**157–177**	nucleic acid binding related	KP279213	8	6.15	1	0.84	0.82
		R:TGATGACTGCGATATGTACTCT	R:55.02								
**311291_K70**	(TC)14	F:CTTGATCTTGTCGCTGTAGAC	F:55.2	**287–303**	udp-d-apiose udp-d-xylose synthase 2	KP279214	7	6.04	1	0.83	0.81
		R:TTCCTTATCGAAATCACGAG	R:55.47								
**313711_K70**	(TC)16	F:CGACTTAATCCCTCTCTTTCTA	F:54.7	**321–340**	beta- -mannosyl-glycoprotein 4-beta-n-acetylglucosaminyltransferase-like isoform x2	KP279215	5	3.98	0.77	0.75	0.71
		R:CTTTACTTTTCCATCTCCCTC	R:54.74								
**313928_K70**	(GA)15	F:CAATTATCAAGGAGGCAATC	F:54.81	**204–224**	photosystem ii 5 kda chloroplastic-like	KP279216	6	3.22	0.85	0.69	0.66
		R:TCACAAATGAGGATCTACACAC	R:55.14								
**314402_K70**	(GA)17	F:TGGAAGAACTCGATACGAAC	F:55.37	**154–170**	30s ribosomal protein chloroplastic-like	KP279217	6	3.41	0.77	0.71	0.67
		R:GAGAAGTTGGATACTGGAAATG	R:55.49								
**314761_K63 ***	(CT)14	F:ATTGTTGGATACTTCATGGC	F:54.99	**137–197**	hypothetical protein VITISV_008952	KP279170	8	5.54	0.85	0.82	0.8
		R:GTTGGTACTGGTAAACCCTAAT	R:54.1								
**314797_K70**	(AC)14	F:CTTGTTCTCCTCCTTTAGTCTG	F:55.05	**201–217**		KP279218	5	3.16	0.85	0.68	0.65
		R:CATCTTCATACTCCTATTGTCG	R:54.14								
**314831_K70**	(GA)14	F:ATCTCTCGTGCCTGTCATAC	F:55.21	**159–169**	syntaxin-71-like	KP279219	4	2.89	0.85	0.65	0.6
		R:CTTTTCGATGTCGTACTTGTC	R:55.05								
**1trimcontig191066**	(TCT)13	F:GATATTAGTCCGGTTTACGAGA	F:55.22	**290–309**		KP279227	5	4.39	0.85	0.77	0.56
		R:GATACAGGAGTCGAGAATGAAT	R:54.99								
**319429_K63**	(GA)17	F:GGAGATAGGAAGTGTGATGAAC	F:55.31	**180–194**		KP279171	6	2.86	0.69	0.65	0.62
		R:TTATTGTGCAAGCATACGAG	R:55.09								

Table S1. *Cont.*

Primer ID	Motif	Primer Sequences (5′-3′)	TM (°C)	Allele Range	Description of Putative Function	GenBank Accession Number	Heterozygosity				
							N_A	N_E	H_O	H_E	PIC
**354699_K63**	(TC)15	F:GAAGCGATTTGGAAGAAAC<br>R:ACACAGAGAGATTACGAACACA	F:54.98<br>R:54.97	**172–191**		KP279172	7	5.12	1	0.8	0.78
**364103_K63**	(TC)17	F:TACAAACCCTAAGCTCTAAACC<br>R:CGACTTGAGTGATACCAAAGA	F:54.68<br>R:55.48	**184–202**	nicastrin isoform x2	KP279173	3	2.3	0.85	0.57	0.47
**372875_K63**	(TC)18	F:CACACACAAATCCCAATTTC<br>R:GATGGTGTTTTCATAGTTCGAC	F:56.29<br>R:56.23	**198–228**	pentatricopeptide repeat-containing protein at1g16830	KP279174	6	3.31	0.77	0.7	0.65
**407841_K63**	(AG)17	F:TTGAGTAGATACATGCTGGCT<br>R:CTCACCCTTTCTCTTGTGATA	F:55.17<br>R:54.52	**274–294**	tyrosine-specific transport	KP279175	6	4.76	1	0.79	0.76
**408825_K63**	(AG)16	F:GTTCTCCTCTTTCATCATTCAG<br>R:AGTCTTGAACTCCTTGTACTCG	F:55.61<br>R:55.39	**262–286**	ankyrin repeat-containing	KP279176	6	3.93	0.92	0.75	0.71
**409500_K63**	(CA)22	F:GATTCCTGGGTGTAGTTCTGT<br>R:CTTAGTCTTTAATGCTGGCTCA	F:55.66<br>R:56.58	**333–395**	pentatricopeptide repeat-containing protein at1g08070-like	KP279177	6	3.48	0.85	0.71	0.68
**409618_K63**	(CT)14	F:CTTCTCCTTCCCTTCACTTTA<br>R:TTAGTGTTAGTGTTGGTGTTGG	F:55.36<br>R:55.37	**249–287**		KP279178	6	5.28	0.92	0.81	0.78
**411145_K63**	(CT)16	F:GGTAGGAATTAAAGTGAAGACG<br>R:ACTAGGGAGCGAGAGAGAGTA	F:54.88<br>R:55.13	**370–389**	tonoplast monosaccharide transporter2 isoform 1	KP279179	6	4.83	1	0.79	0.76
**411348_K63**	(GA)18	F:AATTACCAATGTTCACTCCG<br>R:GTTGATGTAGTTCTGTGGTTGA	F:55.09<br>R:55.23	**266–283**		KP279180	9	5.54	0.83	0.82	0.8
**411475_K63**	(AG)15	F:GCAACAGGGACAGATATTTT<br>R:TACGGACTCATAGAAGGTTAGG	F:54.3<br>R:55.3	**179–199**		KP279181	7	4.39	1	0.77	0.74
**412234_K63**	(AG)17	F:GTGCAAGCCGTTTCTTATG<br>R:ATCGGAGGTTCCATCATTTA	F:56.95<br>R:56.94	**147–155**		KP279182	4	3.41	0.92	0.71	0.65
**414791_K63**	(GA)17	F:ACGACTAGCAGCATTCAGTAA<br>R:CAGGAGATCAGAAAACACAATC	F:55.41<br>R:56.38	**335–362**	PREDICTED: uncharacterized protein LOC104588813	KP279183	8	5.73	0.92	0.83	0.81
**416275_K63**	(TC)15	F:GGTTATGATGGTGGGAAAG<br>R:TGCACCTCCTCTCTCTCTAA	F:55.24<br>R:55.24	**195–197**	probable inactive receptor kinase at1g48480	KP279184	2	1.31	0.27	0.24	0.21

Table S1. *Cont.*

Primer ID	Motif	Primer Sequences (5′-3′)	TM (°C)	Allele Range	Description of Putative Function	GenBank Accession Number	Heterozygosity				
							N_A	N_E	H_O	H_E	PIC
**416328_K63**	(AG)21	F:GTATGCCCAGAATATCCATTAC<br>R:TAGTCACGAGGAAAGCTAAAGT	F:55.25<br>R:55.24	**167–225**	chaperonin-like protein	KP279185	7	4.33	0.77	0.77	0.74
**416815_K63**	(GA)17	F:CGTTTCTTTTCTTCTCTCTCTC<br>R:CCTCCATTCTCTCTCCTACTAA	F:54.43<br>R:54.52	**240–252**	probable receptor protein kinase tmk1-like	KP279186	4	3.1	0.55	0.68	0.62
**417587_K63**	(AG)19	F:TGGGTAGATATTAGATGGCAGT<br>R:CTTCTTCTGGAAATCTGGTTAG	F:55.48<br>R:54.99	**238–275**		KP279187	7	6.26	0.85	0.84	0.82
**417854_K63**	(CT)17	F:AAAAGGAGTCTTGGGAGTAAGT<br>R:TTGAGATGTAACTATGCAGTCC	F:55.39<br>R:54.59	**271–297**	50s ribosomal protein chloroplastic-like	KP279188	7	3.48	0.85	0.71	0.68
**418294_K63**	(AG)18	F:CAAGAACAAGAAGAAGAAGACC<br>R:AGAGACCACCCAAAAGATAAG	F:55.01<br>R:55.07	**336–348**		KP279189	6	4.76	1	0.79	0.76
**418596_K63**	(CT)17	F:CGTGAGTTTGAGTGAGTAATTG<br>R:AGGACATGGTGAGTTGAGAAT	F:55.16<br>R:56.09	**395–401**		KP279190	4	3.48	0.69	0.71	0.66
**418931_1_K63**	(TC)15	F:ATTAGCTCAGTTCCCAGTAACA<br>R:CTTCTTTCTCTTTCTCCTTCCT	F:55.74<br>R:55.33	**165–177**	beta-galactosidase 3	KP279191	5	3.28	0.85	0.7	0.64
**419834_K63**	(TC)16	F:GAAAAGAGAGGAGAAGATGGAT<br>R:TACCAGAACTGTGTGAGATTGT	F:55.75<br>R:54.8	**183–208**	probably inactive leucine-rich repeat receptor-like protein kinase at5g48380	KP279192	6	3.52	0.54	0.72	0.67
**42710_K70**	(AG)16	F:GTTACACACACACCCACAGA<br>R:GAGAGAGGACTAGGTCGTACAG	F:55.12<br>R:54.9	**181–203**	wd-40 repeat-containing protein msi2-like	KP279194	4	3.16	0.85	0.68	0.63
**47166_K70**	(GA)15	F:TATTGAGAGTGTGAGACCGTT<br>R:TGGTAAGTATCGTAGGTCCAAT	F:54.87<br>R:55.4	**310–315**		KP279195	3	1.91	0.69	0.48	0.39
**482_K70**	(CT)15	F:ACAGCGGCATAGTAAAATGA<br>R:GTCACCGAAATCTCACTCAATA	F:56.02<br>R:56.82	**179–192**		KP279193	6	5.45	1	0.82	0.79
**60699_K70**	(CT)19	F:CTTCTCACTGTATTTCTTCGAG<br>R:GGCTACTTTGTTAGGGTAGATT	F:53.65<br>R:53.72	**277–304**		KP279196	11	8.05	1	0.88	0.86
**71002_K63**	(GA)16	F:CTTCAATCCACGAATACCAC<br>R:CAATTATGCAAAGGAGGAAG	F:55.5<br>R:55.07	**235–248**		KP279161	8	4.63	0.85	0.78	0.76

Table S1. *Cont.*

Primer ID	Motif	Primer Sequences (5′-3′)	TM (°C)	Allele Range	Description of Putative Function	GenBank Accession Number	Heterozygosity				
							N_A	N_E	H_O	H_E	PIC
**76126_K63**	(AG)16	F:TTTATTGGAGCGAAAGAGAG	F:54.94	**231–242**		KP279162	5	3.45	0.77	0.71	0.66
		R:AAAAGGGGAGGAGAGAGAT	R:54.38								
**76326_K70**	(GA)15	F:AATGTCTTCCAAATCAGGTG	F:55.03	**279–294**		KP279197	6	5.04	0.85	0.8	0.77
		R:CAAGAACGAACCCTCTATTTC	R:55.63								
**80734_K70**	(TC)15	F:AGGGAGAACCAATTCCTTAC	F:54.81	**347–373**		KP279198	7	5.12	0.92	0.8	0.78
		R:GACCTAACCCTAACCCAGTC	R:55.16								
**82171_K70**	(CT)14	F:TAGTAGAGTTGAAGAGGAGGGA	F:54.97	**168–185**	single-stranded dna-binding mitochondrial	KP279199	6	3.52	0.77	0.72	0.68
		R:CTAGGGTTTAAGCAAGCATAGT	R:54.85								

Note: * = marker displayed segregation distortion $p < 0.05$.

Table 2. Primer characteristics, diversity statistics, and segregation analysis statistics of 412 polymorphic genomic SSRs tested on a diversity panel of 13 cranberry cultivars and 16 progeny from a “Crimson Queen” × “Mullica Queen” cross.

Primer ID	Motif	Primer Sequences (5′-3′)	TM (°C)	Allele Range	GenBank Accession Number	Heterozygosity				
						N_A	N_E	H_O	H_E	PIC
ct106280	(AC)10	F:GCCATAGCTATTTTGTAACGAG	F:55.983	216–225	KP279109	3	2.43	0.69	0.59	0.50
		R:TATCATGGACTAGGTCTCAACA	R:54.829							
ct110752	(TC)14	F:ACACACACTAACGAAATCCTTC	F:55.903	126–141	KP279110	6	4.33	0.85	0.77	0.74
		R:CTAGCTCCGACATTGTTATCTC	R:55.823							
ct115258	(CT)13	F:GTTCGTTGTGGAAGTCACAT	F:55.932	192–207	KP279111	6	4.57	1.00	0.78	0.75
		R:CAAAATGAGTGCCAGATAGTG	R:55.933							
ct116900	(GT)11	F:CTCAAACATACCCTTTGAGC	F:54.959	166–168	KP279112	4	1.91	0.62	0.48	0.43
		R:GGTATAGCTTAACAACACACCA	R:54.579							
ct118602	(TC)9	F:TAGAATGCAGTCGTGAAGTGTA	F:56.18	152–159	KP279113	3	2.66	0.77	0.62	0.55
		R:ACTAAATGAGGGGTAGTACGTG	R:55.091							
ct119523	(AG)11	F:GACTCATGGGAGTGAGGAC	F:55.238	234–279	KP279114	10	6.63	0.92	0.85	0.83
		R:TGAACTTGTGTAGTCTTTACCG	R:54.77							
ct119590	(CT)9	F:ACATGACATCAATTGCCC	F:54.903	186–189	KP279115	4	2.50	0.67	0.60	0.52
		R:TATCCTACCTCAAAGAGCCTAA	R:55.115							
ct120091	(CT)9	F:GTTGAAAGCGACAAGTCTTC	F:55.143	181–183	KP279116	2	1.83	0.39	0.45	0.35
		R:TAATTTTGCCCTACCCACC	R:57.415							
ct121951	(TTGGT)6	F:CATGTAGCCGACTCCAATTA	F:56.296	178–193	KP279117	3	2.05	0.62	0.51	0.46
		R:TATCCCATTCCGTATAAGGTC	R:55.632							
ct124256	(TC)9	F:GCCGTTAGTTCGTGATATGT	F:55.275	209–214	KP279118	2	1.95	0.69	0.49	0.37
		R:CCTACATGCATACGTAAAACAG	R:55.176							
ct129169	(AG)10	F:TAAATCACCTTCTTCCTCCTC	F:55.117	125–133	KP279119	4	3.22	0.77	0.69	0.64
		R:GGTCCCAAACTTACTACTCAAA	R:55.177							
ct129202	(CT)9	F:CGACCTACACGAGATTGTTTAT	F:56.013	276–278	KP279120	2	1.99	0.92	0.50	0.37
		R:GTTCCAAATCTTCAGTAAGCTG	R:55.863							
ct130570	(TTC)6	F:GTTCACAATCTGCATCTCCT	F:55.176	197–200	KP279121	2	1.55	0.31	0.36	0.29
		R:ACGTAATAGATCAAGAACAGGG	R:55.133							

Table S2. *Cont.*

Primer ID	Motif	Primer Sequences (5′-3′)	TM (°C)	Allele Range	GenBank Accession Number	Heterozygosity				
						N_A	N_E	H_O	H_E	PIC
ct132010	(TTTTA)4	F:TACGTGAATTACCCATATCCAC	F:56.448	203–214	KP279122	2	2.00	1.00	0.50	0.38
		R:CTCACCCTTTACTTCTCTTTGA	R:55.449							
ct134336	(TCC)6	F:GAACACTCCTTCTCTAGCTCTG	F:55.183	195–201	KP279123	2	1.55	0.46	0.36	0.29
		R:CTTTTTAGTCTCCGACAATCTC	R:54.98							
ct135942	(TC)12	F:CTACTTGCCTTCCTCTTTGAC	F:55.859	163–170	KP279124	4	3.43	0.17	0.71	0.66
		R:TAAATAATCCGTCCACGAAC	R:55.183							
ct139553	(CT)11	F:GATCAAGCATTGTTCTCTTCC	F:56.45	123–150	KP279125	5	3.35	0.77	0.70	0.66
		R:AGCTATAGGGCTAGCGATG	R:54.943							
ct140233	(GA)14	F:TTACAGAAGGAAGAGAGAGGAA	F:55.096	224–249	KP279126	7	5.05	0.85	0.80	0.78
		R:ACTGGCTTCTATAGCTCATTTC	R:54.707							
ct144370	(TC)11	F:GTAGGAAAAGTTTGAACCGTC	F:55.539	223–237	KP279127	4	3.31	0.77	0.70	0.65
		R:TCAAAGGTTTCACGTTTCTC	R:55.419							
ct144558	(AG)9	F:TCATTACCCCTAACCTCTAAAC	F:54.3	223–227	KP279128	3	2.18	0.69	0.54	0.46
		R:ATTCGACTAGAGTGGAGAGAAA	R:54.961							
ct144936	(AAACT)4	F:AGGTGACTAAGGCAGTGTTC	F:54.398	181–191	KP279129	2	1.95	0.69	0.49	0.37
		R:CGTGTCTGTTTGGTTAGTAGGT	R:56.015							
ct145170	(CTT)12	F:GAATCCTAGCCTATTTCCTTTG	F:56.31	208–223	KP279130	5	3.38	0.69	0.70	0.66
		R:GAAGCAAACACCACTCAATATC	R:56.383							
ct145217	(AT)10	F:CCAGTACTAGATCCACTGCATA	F:54.637	156–165	KP279131	5	3.16	0.77	0.68	0.65
		R:TGTTCTAGAGAGGATGACATTG	R:54.531							
ct145906	(AC)10	F:TCTAGACTTGAGAAGCACTTTG	F:54.272	260–262	KP279132	2	1.55	0.46	0.36	0.29
		R:AGTTAGAGGAGGTTTCTGTTGA	R:54.884							
ct147864	(ATT)6	F:CTCTCTTTACCCTCAATTTCTC	F:54.106	273–276	KP279133	2	1.26	0.23	0.20	0.18
		R:GGTCTAATATCAATCGATGACC	R:55.208							
ct149097	(TCT)6	F:GAACTGACTGAGTCCACAAAAT	F:55.879	257–263	KP279134	2	2.00	1.00	0.50	0.38
		R:GAACAATAGTAACCCATGCAG	R:54.865							

Table S2. *Cont.*

Primer ID	Motif	Primer Sequences (5′-3′)	TM (°C)	Allele Range	GenBank Accession Number	Heterozygosity				
						N_A	N_E	H_O	H_E	PIC
ct152567	(TC)13	F:GTGGCTTTTCTGATCTTGTT	F:54.479	208–226	KP279135	6	4.23	0.85	0.76	0.73
		R:AAAGTACTCTCAATTGGTACGG	R:55.209							
ct153008	(CT)11	F:CTTTCCAAGATCTTCATAGGC	F:55.707	257–266	KP279136	3	2.75	0.77	0.64	0.56
		R:CGACAGTATAATAGCATGGAGA	R:54.741							
ct154206	(CT)14	F:GAGAGCGTACGATACCTAATTC	F:54.976	198–204	KP279137	4	3.22	0.85	0.69	0.63
		R:CTGGTTAGGAAAACCACTAGAA	R:55.337							
ct154615	(CT)9	F:AAAATTGAGCACTGGCTAAG	F:54.889	131–135	KP279138	3	2.00	0.69	0.50	0.41
		R:CTCATACAAACAATAGGGGG	R:54.62							
ct154654	(GA)10	F:GATTTCTAGTGGGAAATGAAGG	F:56.876	167–171	KP279139	4	2.10	0.62	0.52	0.45
		R:GGTGTATGTGTGTGATTAAGGA	R:55.03							
ct155339	(CCA)6	F:AAGTTCCTCTGTTACAAGCTCT	F:54.254	219–228	KP279140	2	1.99	0.46	0.50	0.37
		R:ATGACGAACTCTTCCTCCTTAT	R:56.116							
ct155461	(TG)10	F:GGTTTCAAACTCGAACAAAG	F:55.007	258–277	KP279141	4	2.62	0.85	0.62	0.56
		R:ATCCTATAACTGGGGATAATGC	R:56.239							
ct159707	(CA)9	F:TGTTAGCTCCTTACTTTCCATC	F:55.403	185–188	KP279142	3	1.59	0.46	0.37	0.32
		R:GTGAAGAGGAAGATGAAGAATG	R:55.606							
ct160768	(GTTTG)4	F:GTGTGGTATGTTGGATGTAAAC	F:54.46	139–144	KP279143	2	2.00	1.00	0.50	0.38
		R:TAAGGGGATTTCATTGGG	R:55.759							
ct161908	(TATG)7	F:CCTAGGAGATGGGTCAAGAT	F:55.666	156–173	KP279144	5	2.27	0.62	0.56	0.51
		R:ACCACTGTCTTCCATATTCACT	R:55.263							
ct165512	(CT)9	F:CTTCCTACTCTCTCCCTCTACA	F:54.567	128–139	KP279145	3	2.15	0.31	0.54	0.47
		R:GTTGGATCTTGATGGGTTTA	R:54.967							
ct171223	(TG)9	F:GCGTGTTATTATTCCTCTACCT	F:54.47	152–154	KP279146	2	1.26	0.23	0.20	0.18
		R:GGTACATTCTTTGACCGAGTAT	R:54.968							
ct174735	(TC)14	F:CTTATTTGTATGGCCTTCCT	F:53.652	171–194	KP279147	7	5.28	0.92	0.81	0.79
		R:GCAGCATATATTGTCCAGTTC	R:54.93							

Table S2. *Cont.*

Primer ID	Motif	Primer Sequences (5′-3′)	TM (°C)	Allele Range	GenBank Accession Number	Heterozygosity				
						N_A	N_E	H_O	H_E	PIC
ct188529	(GA)10	F:TTGCAGAATCAATAGTACCTCC	F:56.062	191–232	KP279148	7	2.91	0.85	0.66	0.63
		R:CCTCATTAGCTATGGTGAAAC	R:54.112							
ct89348	(CACCT)4	F:GGCTCAATCTTGTGTAGGTATT	F:55.122	184–194	KP279100	3	1.70	0.39	0.41	0.35
		R:GAGAAAGTGGAAAGATTGTGTG	R:56.452							
ct89379	(TC)10	F:ATGAAGAGCTTGAATGGCTA	F:55.205	171–183	KP279101	5	4.63	0.85	0.78	0.75
		R:ACACTTTACACCACAACTCGTA	R:54.941							
ct89711	(CT)13	F:CTCCACACCCACAATCTG	F:55.585	137–149	KP279102	5	3.13	0.77	0.68	0.63
		R:CGTCTTATTTTTAGTCACCTGG	R:55.749							
ct92708	(TC)10	F:CCCTAGATATTTCTGGAACACT	F:54.261	144–152	KP279103	4	3.98	0.77	0.75	0.70
		R:AAGATAGAGAGAGACAAAGGAGG	R:55.22							
ct93137	(CT)11	F:AAGATTTCCGCTACAGTACCT	F:54.871	177–207	KP279104	5	3.41	0.92	0.71	0.67
		R:GCTATGGGTGTCTCAAAAAG	R:54.959							
ct94504	(AAACA)5	F:CTCTAAAGCTCAAGAAAACGTC	F:55.359	258–268	KP279105	3	1.48	0.39	0.32	0.29
		R:AGCTGTGACTATAAGGGATTTG	R:55.284							
ct95345	(CT)9	F:ACTCTACAAGGGCACGAAC	F:55.289	122–128	KP279106	3	2.09	0.46	0.52	0.44
		R:ATGGAAGTAAGAAAGTGAGTGG	R:55.163							
ct95842	(GA)12	F:GTGGAAAGAGATTGTTGATGTC	F:56.217	222–234	KP279107	5	3.22	0.77	0.69	0.64
		R:AAAACTAATGGATGACGACG	R:55.252							
ct98042	(AAAAT)5	F:CCTTTTAAGTACTTTCCCTTCC	F:55.482	266–272	KP279108	3	1.75	0.54	0.43	0.39
		R:CCCCTCATCTTTATGTGC	R:53.806							
SCF1001	(ACA)7	F:AAACTAGCATATCCCAAGGTAG	F:54.48	209–215	KP278595	2	1.90	0.46	0.47	0.36
		R:ATATAGCAACAGTGGGCAGT	R:54.85							
SCF100820	(TG)10	F:GTAATTCCACTTAACCCACTCA	F:55.83	342–353	KP278804	4	2.97	0.62	0.66	0.60
		R:GTTGAAGATAAACCACCTTCC	R:55.30							
SCF101064	(GT)9	F:CATCAGACAGAAAGCAGTTAAG	F:54.56	296–302	KP278805	3	2.27	0.69	0.56	0.49
		R:CCCCAAGTATATTAGCAAACAC	R:55.55							

Table S2. *Cont.*

Primer ID	Motif	Primer Sequences (5′-3′)	TM (°C)	Allele Range	GenBank Accession Number	Heterozygosity				
						N_A	N_E	H_O	H_E	PIC
SCF101878	(CAA)7	F:GACTCATTGGATACGTGCT	F:53.42	343–346	KP278807	2	2.00	0.54	0.50	0.38
		R:TCTATGTAGCTTTGAAGTGAGG	R:54.10							
SCF101914	(TC)10	F:CTTTGGAGCACAACAACTCTA	F:55.72	150–165	KP278806	4	3.17	0.75	0.68	0.62
		R:GTGTAAAGACCAGGACCCTT	R:55.65							
SCF102347	(AG)12	F:GGTAGTGAGCAACGACATAAC	F:54.91	335–349	KP278808	7	4.94	1.00	0.80	0.77
		R:CCTGAAGGTAAAGAAAGTAGCA	R:55.63							
SCF10459	(TC)11	F:TCTTTGTTTCTGAGGTTGCT	F:55.15	253–257	KP278631	3	2.36	0.77	0.58	0.50
		R:ATTTGTAGGTACTATGGAAGCC	R:54.32							
SCF104688	(GA)10	F:ACAAAGAAATGTATGGCACC	F:55.7	194–207	KP278809	5	3.27	0.92	0.69	0.49
		R:CTTTTCGTCTCCTCTAATTCC	R:54.89							
SCF1047	(AC)10	F:GAGCTTTGGCCTCATATTACT	F:55.33	266–267	KP278596	3	2.27	0.85	0.56	0.49
		R:CGAATTACTCCAACCAACAT	R:55.8							
SCF105092	(AT)11	F:AGGAACTAGGAAGTAGGAAGATG	F:55.11	140–176	KP278810	5	3.89	0.31	0.74	0.70
		R:GTGCTATACAGGCATACAAGTG	R:55.15							
SCF10514	(GA)11	F:GTACTCTTTGTCGGATGTTTTC	F:55.55	240–252	KP278632	4	3.22	1.00	0.69	0.63
		R:GTTTCACTCCCACCTCTTAAT	R:54.90							
SCF105151	(GA)9	F:CAGAATAAGATTGGGTAGAAGG	F:54.81	284–286	KP278811	2	1.60	0.50	0.38	0.31
		R:TTTGAGAATTACTTGGCACC	R:55.38							
SCF105925	(TC)15	F:CCGTGTCAAAAGATCAAGC	F:56.80	159–166	KP278812	4	2.70	0.62	0.63	0.57
		R:AGTTTGTGCCGTCGTACTC	R:56.29							
SCF106182	(GT)11	F:TACCCTTGTGTATCCCTACATT	F:55.25	151–153	KP278813	2	1.74	0.46	0.43	0.34
		R:GAACAATAGCAGCAACAGAAC	R:55.20							
SCF107429	(CT)13	F:ATGTGAGGTGGGATGATATTAG	F:55.56	348–378	KP278815	9	7.51	0.92	0.87	0.85
		R:ATATGGTGTCAGTGTGGTGTAG	R:54.96							
SCF107477	(AG)10	F:GTCTTATTTTCACTGTCGTGTG	F:54.98	190–200	KP278814	5	2.64	0.77	0.62	0.56
		R:CGGGCATTAACCTTATACCT	R:55.87							

Table S2. *Cont.*

Primer ID	Motif	Primer Sequences (5′-3′)	TM (°C)	Allele Range	GenBank Accession Number	Heterozygosity				
						N_A	N_E	H_O	H_E	PIC
SCF107715	(AG)11	F:AAAGCGAGTCAGAAACATAGAC	F:55.46	244–302	KP278816	7	4.69	0.92	0.79	0.76
		R:CCTATCAGTTCCTTTCCTATTG	R:54.81							
SCF10785	(TC)10	F:ACATAAAGGAGAGGGAGTAGAG	F:53.58	247–251	KP278633	3	1.49	0.39	0.33	0.30
		R:ATACCACTTGATAGATTCCTCC	R:54.0							
SCF108252	(TC)10	F:CCTATGTAATTGGATTCTACCC	F:54.48	204–208	KP278817	3	2.33	0.77	0.57	0.50
		R:GTGTATCAAGGTGGAGAAAGTC	R:55.38							
SCF108294	(TC)12	F:GGTAAGATTGAGGTTCTGGTCT	F:56.44	240–260	KP278818	4	3.05	0.92	0.67	0.62
		R:GGTAGAAGCAAGAAGATGCAC	R:56.70							
SCF108454	(TG)9	F:CTAACTAAATGAAGTGTTCCCCT	F:55.81	192–198	KP278819	4	2.50	0.62	0.60	0.52
		R:ATGTCACGCTCTGAAGTTTG	R:56.45							
SCF109269	(TC)11	F:CACTCCTTCCTTATAGATCAGC	F:54.93	242–268	KP278820	5	2.27	0.69	0.56	0.51
		R:AAGTAGAAGAGCAGCACAAGAG	R:55.85							
SCF109660	(GA)9	F:CCCCAAACTGTCGTATAAAA	F:55.27	289–291	KP278821	2	2.00	0.54	0.50	0.38
		R:TAGAGTACAGGAAAAGCCCTAA	R:55.18							
SCF110168	(GA)12	F:AAAGGACTAGAGGGAAGTACAAC	F:55.3	316–347	KP278822	7	4.39	0.92	0.77	0.74
		R:CTTATTATCCAGAAACTCGTGC	R:55.81							
SCF110223	(GA)11	F:GATTCTGTTCCAATAGGCATAC	F:55.43	347–371	KP278823	6	5.04	1.00	0.80	0.78
		R:GGAGTAGTAGTGAAAGGACCAA	R:55.16							
SCF110507	(CT)14	F:GTAGCTGAGGTGGAGGATAAC	F:55.49	221–235	KP278824	5	3.89	0.92	0.74	0.70
		R:GAGCTGGTGCTGAAATTAAC	R:55.11							
SCF11065	(TC)11	F:CTTTGTCCCAACACGTTAAT	F:55.17	191–193	KP278634	3	1.50	0.40	0.34	0.30
		R:AAGTCTATAAGCATCCTGCAAC	R:55.42							
SCF110757	(GA)9	F:TCATATCAACCTAACAATCGG	F:55.20	286–342	KP278825	6	1.82	0.46	0.45	0.43
		R:CACAAACAAGGAAATTAAGACC	R:55.56							
SCF11084	(CT)9	F:GTTGGCTGAGGTAGCTGATAG	F:56.74	312–316	KP278635	3	1.61	0.46	0.38	0.34
		R:CCTAAAAGGGCTCACAAGTTA	R:56.27							

Table S2. *Cont.*

Primer ID	Motif	Primer Sequences (5′-3′)	TM (°C)	Allele Range	GenBank Accession Number	Heterozygosity				
						N_A	N_E	H_O	H_E	PIC
SCF110888	(CA)9	F:CTCCTACCCAAATTCACTTGT	F:55.82	191–193	KP278826	2	1.35	0.31	0.26	0.23
		R:CCAAAACTAAACCATTTCTCAC	R:55.56							
SCF111145	(TA)9	F:TTAGTCTGGCTGGTTTTAGTTT	F:55.10	342–350	KP278827	5	4.08	0.60	0.76	0.72
		R:TTGTACCTATTGTTGGATTGTG	R:55.21							
SCF111370	(AC)14	F:ACCACATCTTCATTTTGAGC	F:55.19	273–284	KP278828	6	3.71	0.92	0.73	0.69
		R:GTAAAACAATACGGGTCCTTAC	R:54.63							
SCF11186	(TCC)9	F:AGAAAGGCTAAAAGGGTATCTC	F:55.18	278–287	KP278636	2	2.00	0.54	0.50	0.38
		R:GCTCTCAACAACTCGAAAGTA	R:54.92							
SCF112295 *	(AC)9	F:AACATCTCTACCTCTCACGTTT	F:54.74	271–273	KP278829	2	1.08	0.08	0.07	0.69
		R:TAGTATTAGTTGATTTGGCGTG	R:54.84							
SCF112540	(CT)9	F:CAGTAGTGGTATTTCACAATCG	F:54.97	224–226	KP278830	2	1.26	0.23	0.20	0.69
		R:TTTAATGCTTTTGGAAGAGG	R:54.80							
SCF1128	(AG)13	F:GTTTGTTGTTGTGGTGGTTT	F:55.83	304–322	KP278597	5	3.77	1.00	0.74	0.69
		R:CCTTACTTGACGCTTACTTCAG	R:56.12							
SCF113389	(AC)9	F:GACATCACTCAAGCAAGATAAA	F:54.71	157–171	KP278831	5	2.70	0.77	0.63	0.58
		R:CCTCGATTCCTCAAGATATG	R:54.8							
SCF113558	(TTC)11	F:GAGCTTGATCTGGGTATCTTT	F:55.13	200–206	KP278832	4	2.25	0.39	0.56	0.51
		R:CAAAATCAGAATCGACTGC	R:54.24							
SCF11431	(TC)13	F:GCTGCTGATTTGTTATGTAGAG	F:54.53	306–318	KP278637	5	2.79	0.85	0.64	0.60
		R:CACTTAGCCCCTTAAACTATTG	R:55.8							
SCF116329	(AT)12	F:GAATCCCACATTAGAAGTTGAT	F:54.85	191–199	KP278833	4	2.62	0.31	0.62	0.56
		R:TTGTATCTTCCCTATTCCTACTG	R:54.47							
SCF116485	(CAT)7	F:CAATATAAACGTCAGTCACCAG	F:54.97	226–232	KP278834	2	1.95	0.69	0.49	0.37
		R:ACTTTTGGTTATGCTGGAAG	R:54.56							
SCF116567	(GAT)8	F:GTTGGTCTACAATTCTGTTCCT	F:54.99	201–207	KP278835	3	2.75	0.85	0.64	0.56
		R:GCCCTTTTAGTTGAAATGC	R:55.3							

Table S2. *Cont.*

Primer ID	Motif	Primer Sequences (5′-3′)	TM (°C)	Allele Range	GenBank Accession Number	Heterozygosity				
						N_A	N_E	H_O	H_E	PIC
SCF116864	(AC)9	F:TGCCCCTTGATTCTAATTTT	F:56.35	150–154	KP278836	3	2.60	0.77	0.62	0.54
		R:ATGCCTCAGATTGATTTACCT	R:55.42							
SCF117157	(GA)15	F:GGATAGAAACCTGATACGGAC	F:55.26	194–204	KP278837	6	5.05	1.00	0.80	0.77
		R:CGTTACCGTCCCAAATATAA	R:55.24							
SCF117385	(GA)12	F:TAAGAATCCTCGTCATAGGGT	F:55.43	145–153	KP278838	4	2.40	0.77	0.58	0.51
		R:CTGTCTTCTCAACTTTCCCTC	R:55.67							
SCF117422	(TC)12	F:TTCTGTTTCTTGGCTCTGTATC	F:56.26	249–275	KP278839	7	3.76	0.77	0.73	0.69
		R:TATTATGCTACATCGGTCGAG	R:55.55							
SCF11802	(AAC)8	F:CGAGGAACAAGTTTTATAGGAG	F:55.4	290–293	KP278638	2	1.99	0.62	0.50	0.37
		R:ACACTCACCTTTATTATGGGAC	R:54.81							
SCF118468	(CT)15	F:ATAAGCGGAGCACAGTTACA	F:56.10	242–260	KP278840	6	3.49	0.92	0.71	0.67
		R:GATAGGATGACCTGTTTTGGT	R:55.58							
SCF118608	(TC)14	F:AACTACTCGATCTTCACCCTTA	F:54.86	239–256	KP278841	6	4.45	0.92	0.78	0.74
		R:AGGAGACCAACACTTAACCTC	R:54.96							
SCF118999	(AG)9	F:CTAAACTCCAAAATGCCTAAAC	F:55.4	272–313	KP278842	5	2.06	0.62	0.52	0.47
		R:AAAGTGGATGGGTTCTAAAAG	R:55.8							
SCF120352	(GA)10	F:AGTTCTATGACCCCTAACTGAA	F:54.70	272–295	KP278843	5	3.89	0.69	0.74	0.70
		R:GAAAGGAAAGAAGCACTATCAC	R:54.96							
SCF120937	(GA)13	F:TGTGCAAGAGTCATCTCCTAT	F:54.94	285–305	KP278844	6	4.76	0.92	0.79	0.76
		R:TATTCCCTTTCATTCTCCTTC	R:55.5							
SCF121995	(AC)14	F:TAGTCGTGACCAAGAGTGATTA	F:55.7	169–179	KP278845	4	3.63	0.85	0.73	0.67
		R:GCCACCGAGTATATTTCTATGT	R:55.9							
SCF122746	(TC)13	F:ATTGTATGAAAACCCTAACCC	F:54.74	191–218	KP278846	7	4.33	0.85	0.77	0.73
		R:GAGACGATTCCAAATATAGCA	R:54.59							
SCF123189	(TC)9	F:CCTAGAAATGTTACTCTCCGAC	F:54.86	191–193	KP278847	2	1.74	0.62	0.43	0.34
		R:TTCACTTCCTTACTCCTTTCAT	R:54.82							

Table S2. *Cont.*

Primer ID	Motif	Primer Sequences (5′-3′)	TM (°C)	Allele Range	GenBank Accession Number	Heterozygosity				
						N_A	N_E	H_O	H_E	PIC
SCF124075	(GA)13	F:ATTTTCCCTCCAACCTCTAT	F:54.34	344–362	KP278848	6	4.69	0.85	0.79	0.76
		R:GGTGCAACCAACTAACATAA	R:53.7							
SCF124322	(TC)9	F:TAAAACTGTGAGGTTCAATGTG	F:55.41	214–235	KP278849	5	4.17	0.92	0.76	0.72
		R:CTTCGTGTCTCAAATTACAAAA	R:54.83							
SCF124927	(TG)12	F:CGAGTGTCATTAGCAACAGA	F:54.92	230–236	KP278850	4	2.89	0.85	0.65	0.60
		R:TATCACTTTAGATCGAGCAGAC	R:53.99							
SCF125251	(GA)21	F:TATACAGTCAGATCCAATCCAC	F:54.16	240–267	KP278851	7	5.28	1.00	0.81	0.79
		R:TGCAGATAAAGTACAAGAGTGC	R:54.99							
SCF125667	(CA)10	F:AAGGGAGACATTACACAACAA	F:54.72	171–188	KP278852	7	4.45	1.00	0.78	0.74
		R:TTCGAGATTGACCAAGTATGT	R:54.80							
SCF125889	(GA)17	F:TCTCGTGTATTTTGGAGTGA	F:54.19	175–195	KP278853	6	4.97	0.92	0.80	0.77
		R:GTTGTATCCTTTGTCGATTCT	R:53.47							
SCF126708	(CA)9	F:CGACGAATAAACAAATCAAGTA	F:54.81	315–317	KP278854	2	1.95	0.54	0.49	0.37
		R:GAGAAGAAGTGAAGGAGAGTTG	R:54.98							
SCF127023	(TC)14	F:TATGCTAATCCACTTTGTAGGG	F:56.8	200–212	KP278855	5	3.67	0.92	0.73	0.69
		R:AATCTGGGTAATTGGGAACT	R:55.9							
SCF128015	(TCT)7	F:ACCCACTCTTTCTATTATCTTCC	F:54.90	216–219	KP278856	2	1.90	0.62	0.47	0.36
		R:GTGAGTTCCAAGTTCCACATA	R:55.13							
SCF128307	(TG)14	F:ACTCAGAAGTTGAAGCACAAA	F:55.25	220–262	KP278857	11	6.15	1.00	0.84	0.82
		R:GTATCAAGTACACCAACACCAG	R:54.69							
SCF128992	(TC)11	F:GAGTGTTGAGTTATAGGGGTTT	F:54.17	237–239	KP278858	2	1.90	0.31	0.47	0.36
		R:TCACAAGAATAGAAGGATGGA	R:54.86							
SCF13006	(GCA)7	F:AAAACATAAGAAAGAGCCCC	F:55.14	300–303	KP278639	3	1.81	0.46	0.45	0.38
		R:GGATGATGATGTATGGGAAT	R:54.47							
SCF131915	(AT)9	F:TTTTGTTTCCTTATTTTCGG	F:54.67	184–223	KP278859	6	4.65	0.83	0.79	0.75
		R:TGTAAGTGCATGAAATCGTAAT	R:55.5							

Table S2. *Cont.*

Primer ID	Motif	Primer Sequences (5′-3′)	TM (°C)	Allele Range	GenBank Accession Number	Heterozygosity				
						N_A	N_E	H_O	H_E	PIC
SCF132369 *	(TTC)14	F:CTACTTTGGGATGGAGAGAGTA	F:55.25	261–292	KP278860	8	5.83	0.85	0.83	0.81
		R:AGGTTTAGGTAGTGTTGGATTG	R:55.5							
SCF132506	(TCA)10	F:AATGTGCCAAGTTTTGTAGAC	F:54.48	270–282	KP278862	3	2.30	0.85	0.57	0.47
		R:GTCCCCTATAAGTCATCTGAAA	R:55.2							
SCF132532	(AG)12	F:GACTGGATTTTCACGAATCTAC	F:55.47	275–285	KP278861	4	2.30	0.77	0.57	0.50
		R:CTTCATCTTCCTTGACACTTCT	R:55.27							
SCF132595	(AC)13	F:CAAACAAATCTCAACAACACC	F:55.64	222–277	KP278863	7	4.97	0.92	0.80	0.77
		R:ATTTCAAGATAAGCTCTCCACC	R:56.64							
SCF132922	(AT)12	F:TTAGACGCTTTATGTCCATTC	F:54.68	199–256	KP278864	7	4.69	0.62	0.79	0.76
		R:GAGTGTCCTTGTCTTTGTTGTA	R:54.52							
SCF133376	(AAG)9	F:ATTAGCACCGAATTTAACACC	F:55.91	238–244	KP278865	3	1.62	0.46	0.38	0.35
		R:GATTATGGGTGAGTCTGTGAAT	R:55.59							
SCF136207	(CT)9	F:GTCTCTGTAGTCGGTGCTTT	F:54.57	167–171	KP278866	3	2.50	0.77	0.60	0.53
		R:GATTTCGATTCCTTGACACT	R:54.21							
SCF136317	(GA)10	F:GAGAGTTCAAATTACCTGTACCA	F:55.18	271–277	KP278867	3	1.49	0.39	0.33	0.30
		R:GGAGATTAGGTTGTGGACTAGA	R:55.8							
SCF136826	(CA)9	F:GATCTTGATTAGCTCCAACTG	F:54.19	267–269	KP278868	2	1.99	0.31	0.50	0.37
		R:GCTTACACCAATTCTACAGTCA	R:54.67							
SCF13711	(GAG)7	F:GACTTCCTTGGTACTTGGTG	F:54.68	348–354	KP278640	3	2.77	0.92	0.64	0.57
		R:ACTTTGAGGGTAGGAGTAAACA	R:54.78							
SCF137494	(CT)10	F:CCAACATAAAGAGGACTAGAGG	F:54.87	336–397	KP278869	3	1.98	0.31	0.49	0.43
		R:GACCTAGACTCCAAATCACG	R:54.76							
SCF13753	(CT)9	F:AAGTCCTTTCCTTCTTTTGC	F:55.33	195–199	KP278641	3	1.81	0.54	0.45	0.38
		R:GCTATGTGATGTCGTTCCTAA	R:55.42							
SCF13771	(GA)11	F:AGGATGATGAAATCTGCAAG	F:55.28	171–191	KP278642	6	3.60	0.92	0.72	0.68
		R:ATCAGTTAGGTGGGGTAAGG	R:55.61							

Table S2. *Cont.*

Primer ID	Motif	Primer Sequences (5′-3′)	TM (°C)	Allele Range	GenBank Accession Number	Heterozygosity				
						N_A	N_E	H_O	H_E	PIC
SCF138014	(TC)11	F:TTATTCTCTTCGCTTGGGTA	F:55.22	243–245	KP278870	2	1.45	0.39	0.31	0.26
		R:TCAGATCATGGATTACTGGTT	R:54.54							
SCF138394	(GA)11	F:AAGCCCAGAAGAAATAACCTA	F:54.94	218–229	KP278871	4	3.31	0.83	0.70	0.37
		R:TGCAAATGTTAGGAACTGTGT	R:55.83							
SCF138607	(GA)17	F:CATATAGAATACTGGACGGACA	F:54.42	203–215	KP278872	4	3.10	0.77	0.68	0.63
		R:TTCTGCCATCTCCTTTCTC	R:55.40							
SCF138992	(GA)11	F:ATACTTTACCCCACAGAGCTTA	F:54.91	227–229	KP278873	2	1.65	0.23	0.39	0.32
		R:CCACTCATGCTCACATCAC	R:55.78							
SCF139334	(GT)10	F:GAGGGTCTAATATCTGGTTTCA	F:55.2	203–205	KP278874	2	1.08	0.08	0.07	0.07
		R:GAGAAAAGATGGAGCAAAAG	R:54.31							
SCF139660	(GA)12	F:ATAAATCTACGTCCATACAGCC	F:55.9	333–379	KP278875	6	4.45	0.92	0.78	0.74
		R:GAGTACATACAAATCCTCTTTCG	R:54.89							
SCF140628	(GT)10	F:GTGAAATTGGTCAGGTTGAT	F:54.84	143–154	KP278876	3	2.70	0.77	0.63	0.56
		R:GTCGTCATCATCATCTCCTC	R:55.35							
SCF14119	(TC)9	F:TAACAGTACAATGCCTAGTTCG	F:54.90	243–245	KP278643	2	1.26	0.23	0.20	0.18
		R:GGATTCTCTTGCTTTGGTATAG	R:54.95							
SCF141794	(AAG)7	F:CCATCTGCATCTATTGTTTTG	F:55.84	149–164	KP278877	4	2.27	0.62	0.56	0.47
		R:CATTTGTAGGTCTATCTTTCGC	R:55.81							
SCF14189	(TC)9	F:GTCTAGGTGAGGATGGTTGAT	F:55.58	263–280	KP278644	2	1.35	0.31	0.26	0.23
		R:AAAACAGAGCCCAACAAGT	R:54.82							
SCF141985	(AG)11	F:GAATGGTCTTGAGGGATGTAT	F:55.50	180–188	KP278878	5	4.12	0.85	0.76	0.72
		R:ACTCTGGAAGAAATAAAACGG	R:55.24							
SCF142441	(TC)10	F:TTGCGTTTACTATCTAAGGAGG	F:55.59	219–233	KP278879	5	3.63	0.92	0.73	0.68
		R:CTCAGCCGTCCAAAAGTAT	R:55.39							
SCF142664	(AG)9	F:TACTGACGATGAGCTAGAGTTG	F:54.98	247–249	KP278880	3	1.86	0.62	0.46	0.40
		R:AATGACAAGTGGAATAGTAGGC	R:55.12							

Table S2. *Cont.*

Primer ID	Motif	Primer Sequences (5′-3′)	TM (°C)	Allele Range	GenBank Accession Number	Heterozygosity				
						N_A	N_E	H_O	H_E	PIC
SCF142767 *	(TC)10	F:ATAGTTGGACGGGTGTAATG	F:54.95	257–293	KP278881	8	4.51	0.92	0.78	0.75
		R:CTCTCGCAAAGTAGAACAATCT	R:55.62							
SCF143035	(AT)15	F:TATTTATAGACGACCAACCTGC	F:56.7	166–208	KP278882	6	5.28	0.77	0.81	0.78
		R:GTGACCAATATACCAAACCAAG	R:56.10							
SCF143318	(GA)12	F:CCGTGCTTAAATTCTGTAGTG	F:55.27	279–300	KP278883	5	3.63	0.92	0.73	0.68
		R:TCATCCATAGGAGAACATCC	R:54.85							
SCF144748	(TC)10	F:ATTTCCAATCCTTTCCTCTC	F:54.85	151–153	KP278884	2	1.90	0.62	0.47	0.36
		R:CTCTGACACCTTCTGACACATA	R:55.39							
SCF145689	(TC)13	F:GGCATAAGAGTAGACCATGAAC	F:55.50	260–277	KP278885	5	2.91	0.69	0.66	0.59
		R:GTACTATAAAATGCTTCCAGCG	R:55.98							
SCF145739	(CT)10	F:AAATCCTCCTGTTTTAGACTCC	F:55.69	240–242	KP278886	2	1.99	0.46	0.50	0.37
		R:CCTCAAGTCATCATTCCCT	R:54.91							
SCF146740	(AC)15	F:ATGGGACTGCTTATTGAACAC	F:56.67	202–222	KP278887	6	3.52	0.85	0.72	0.68
		R:CAAGTGGTGCATTGTGAGA	R:56.54							
SCF147117	(TC)9	F:AGATATGGAGTGGATTAGGTTG	F:54.90	240–246	KP278888	3	1.49	0.23	0.33	0.30
		R:GTTAGAGTGAAATGAGCCCTAT	R:54.40							
SCF147295 *	(GA)9	F:ACTGAGGTAAAAGAGGAGTACG	F:54.54	199–207	KP278889	3	2.45	0.85	0.59	0.52
		R:CCATCAAGGTCTCAATCTGT	R:55.1							
SCF147358	(AG)10	F:GTACACTAAACACCTTGCGTTA	F:54.81	211–213	KP278890	2	1.74	0.62	0.43	0.34
		R:CTCACCTACATCCCTCTAGTTC	R:54.67							
SCF149633	(GA)14	F:CCTTAATACCCATCCCATAATC	F:56.46	289–310	KP278891	8	5.54	0.92	0.82	0.80
		R:CTTCTTTTCATTGTTGTGGC	R:55.87							
SCF149976	(TTC)19	F:TATACCCATGTATGTACGCATC	F:55.13	272–309	KP278893	8	6.26	0.92	0.84	0.82
		R:ACTCTAAGCAGGACAATGCTAT	R:55.3							
SCF149989	(AG)11	F:AGTAGGCATTGTTCACTCACTC	F:56.7	293–302	KP278892	5	1.98	0.62	0.49	0.47
		R:TTTCTCCTAAAGCTAAACTCCC	R:56.12							

Table S2. *Cont.*

Primer ID	Motif	Primer Sequences (5′-3′)	TM (°C)	Allele Range	GenBank Accession Number	Heterozygosity				
						N_A	N_E	H_O	H_E	PIC
SCF150173	(AG)12	F:GTGTTGGGAAACAGCAGAT	F:56.1	182–186	KP278894	3	2.07	0.77	0.52	0.42
		R:TTATTCTCGTTGTCAGCCTT	R:55.12							
SCF150898 *	(AG)11	F:AAGCTCCATGTATGCGTATC	F:55.35	311–317	KP278895	3	2.05	0.62	0.51	0.46
		R:ACACTGACTAGCGTTTGTTGT	R:55.6							
SCF150919	(AT)10	F:TTGTTAGCACTTAGCATAACCC	F:56.28	333–363	KP278896	5	4.23	0.85	0.76	0.72
		R:GCTTCATCTCCACCAATACAT	R:56.60							
SCF1527	(GA)9	F:TCAAACGGTGACATCTATACAC	F:55.19	241–256	KP278598	4	2.44	1.00	0.59	0.50
		R:GTATCTACGCCTCTTACTCTCG	R:55.19							
SCF153094	(TC)9	F:TGTCATTAGGGTTCCTCAAA	F:55.64	210–235	KP278897	8	3.45	0.69	0.71	0.69
		R:CACCTAGACAACATCGAAACTA	R:54.68							
SCF153636	(TC)14	F:GGTATCAAAGCAAGGTTGAG	F:54.95	275–297	KP278898	6	4.57	0.85	0.78	0.75
		R:CTCGTTAGAAGTATGTTGGTGA	R:54.68							
SCF153722	(TC)11	F:AGTTATGAGGCTTACGAGGAG	F:55.41	263–279	KP278899	4	3.89	0.92	0.74	0.70
		R:GATGGAACGATGAAACTGAT	R:54.93							
SCF154541	(GA)12	F:AGAAAGCACAGTAGGTATGGAG	F:55.35	265–285	KP278900	7	5.12	1.00	0.81	0.78
		R:CAAGAAACCCTAGAGACCAAT	R:55.7							
SCF155637	(AT)13	F:TGTTAGTGTTAGGACCCGTTA	F:54.95	206–224	KP278901	6	5.12	0.85	0.81	0.78
		R:AAAGTAGGAGTTAGGATGGGAT	R:55.13							
SCF155797	(TC)9	F:ATCATTAAGGCTCCCAAAG	F:54.37	187–188	KP278902	2	1.83	0.54	0.45	0.35
		R:GTACGTCTACTCTGACGGCTA	R:54.83							
SCF156807	(CA)9	F:AGGAGGTTTGGACTAGAAGTTT	F:55.38	150–161	KP278903	4	2.33	0.31	0.57	0.48
		R:CCTGGTTGTCGGATTAGAT	R:54.97							
SCF157992	(TTG)9	F:TAGGTTTGTCTCTTATCCATCC	F:55.2	266–272	KP278904	3	2.27	0.62	0.56	0.49
		R:GAGTTTGTGATTTCTTAGGAGC	R:54.96							
SCF158255	(AG)15	F:ATGCGTACACCTCAATCTTT	F:54.81	300–303	KP278905	4	3.45	0.11	0.71	0.66
		R:GTGGGTACTTGTTTTCAGTTC	R:53.85							

Table S2. *Cont.*

Primer ID	Motif	Primer Sequences (5′-3′)	TM (°C)	Allele Range	GenBank Accession Number	Heterozygosity				
						N_A	N_E	H_O	H_E	PIC
SCF15845	(AG)10	F:AGGCTAATGAAGAAGAAGTCTG	F:54.74	312–328	KP278645	4	2.70	0.77	0.63	0.57
		R:GACCAAGACAAGATGAACAAG	R:54.82							
SCF158633	(GA)18	F:AGATGCTGAAGTTTTCCCTT	F:55.60	168–195	KP278906	8	4.72	1.00	0.79	0.76
		R:TATGTGGATTCTTTGCCTTG	R:56.26							
SCF158988	(TC)10	F:CTCTCACCAAAATCACCATTAG	F:56.39	228–264	KP278907	8	5.45	0.92	0.82	0.79
		R:CAAGTATCAAGTTTTAGACGGG	R:55.74							
SCF159195	(CT)9	F:AACAAAGACCCTAATCAGACAC	F:54.99	309–315	KP278908	3	1.91	0.54	0.48	0.39
		R:ACAATCAAAACACCGTCAG	R:54.35							
SCF160647	(GA)10	F:TAACTCAAAGAACCTAACCCC	F:55.8	162–178	KP278909	5	3.28	0.85	0.70	0.65
		R:TAAAGTGACAGGTAATGTCGTC	R:54.51							
SCF160663	(TC)10	F:TTACACCCTATCTCCTGTTTTC	F:55.1	149–155	KP278910	4	3.05	0.77	0.67	0.62
		R:CAGTTCATCTTGCTAGTTATGC	R:54.53							
SCF16166	(AC)15	F:CCTAGTCATTCTTCTACTCCCA	F:55.25	307–322	KP278646	7	5.63	0.92	0.82	0.80
		R:GGGTTATCTCGTCCATATTGT	R:55.54							
SCF161998	(GT)9	F:ATATACCAGTGCTCTTTCCATC	F:55.4	273–275	KP278911	2	1.55	0.31	0.36	0.29
		R:AGACTTCTTTCTCCAAAGGC	R:55.32							
SCF162175	(TC)11	F:ACACGTTGAGGTTCCAAAT	F:55.38	175–179	KP278912	3	2.02	0.54	0.51	0.45
		R:AGTTTCTGATTGACCTAGATGG	R:55.8							
SCF162565	(GTA)10	F:CTTCCGTGATTGTTCTTGTAG	F:55.6	155–180	KP278913	5	4.23	0.85	0.76	0.73
		R:ACACAGATGGGATGTTGTATC	R:54.72							
SCF163134	(AG)12	F:CAGTGCAATTAGTTTCCTATCC	F:55.66	224–233	KP278914	5	2.58	0.62	0.61	0.57
		R:TTCTTGGGTTGGTTATTCAG	R:55.22							
SCF16359	(AG)12	F:GAAGTGCTTTTCTTTCGTAGAG	F:55.35	316–328	KP278647	3	2.45	0.54	0.59	0.52
		R:AGACAGATTAAGATCCACCTTG	R:55.8							
SCF16407	(TC)11	F:GGCAGTGAATTAAAGGTCAAC	F:56.37	271–279	KP278648	3	2.09	0.62	0.52	0.35
		R:GATGAGAAAGAAGAGTAAGGCA	R:55.51							

Table S2. *Cont.*

Primer ID	Motif	Primer Sequences (5′-3′)	TM (°C)	Allele Range	GenBank Accession Number	Heterozygosity				
						N_A	N_E	H_O	H_E	PIC
SCF1648	(TG)10	F:GTTGATCTGAAGGAAACCAA	F:55.16	284–288	KP278599	2	1.83	0.54	0.45	0.35
		R:TCGTATTAACTCCCCTATTGAC	R:55.7							
SCF164915	(AC)12	F:CTCAAAGTATCTCACTCACGC	F:55.20	191–211	KP278915	6	5.54	0.85	0.82	0.79
		R:ACTGTTGTCCCCTCTGACTAC	R:55.74							
SCF167793	(GA)9	F:GTGAAACGACAAGACCAAAT	F:55.10	180–184	KP278916	3	1.59	0.46	0.37	0.32
		R:AGGACATCCACCTTCAAAT	R:54.29							
SCF169090	(TG)10	F:GAGACAAAGTTCAAATAGGGAG	F:54.82	249–258	KP278917	4	2.94	0.77	0.66	0.60
		R:ATACTGCAACCGATACTGAGA	R:55.0							
SCF170213	(TC)13	F:GGGTTTGATGACTTGTTTGTA	F:55.14	158–166	KP278918	4	3.07	0.69	0.68	0.61
		R:CCTAGAAAATGCAGAAATCG	R:55.22							
SCF171621	(CT)12	F:CACCACTCCCCATTTTAAG	F:55.50	196–209	KP278919	6	3.13	0.92	0.68	0.64
		R:AAGGGACAGAGGAAGTATTTG	R:55.7							
SCF172019	(GA)14	F:TGTGAGTAGTTGTTGAAGGGA	F:55.82	244–279	KP278920	5	4.39	1.00	0.77	0.74
		R:CCTCGAAAATCCGGTAAAT	R:56.64							
SCF172027	(CCA)9	F:ACTCCTATTGCCATTCCAC	F:54.95	161–173	KP278921	3	1.26	0.22	0.20	0.19
		R:CAGTGACAGAGTTGTGGTTAAG	R:55.6							
SCF172149	(CT)9	F:GTTAAATGATGCTGTTAGGGAG	F:55.66	187–205	KP278922	5	3.41	0.77	0.71	0.67
		R:ATGTCCAGTCGTTATCTCTAGG	R:55.11							
SCF172906	(GA)10	F:CTGTTCAAGGATTTGTACTGG	F:54.90	167–179	KP278923	3	2.31	0.44	0.57	0.49
		R:TATTGACATGAGAAGCACGA	R:55.29							
SCF173212 *	(TC)9	F:TGTAGTGGGAGATGCTGATAC	F:55.25	198–206	KP278924	4	2.24	0.69	0.55	0.50
		R:AATTGGCGAACTAGAAAGTG	R:54.72							
SCF174394	(CT)9	F:GGTGGATGGAATGCTAAATA	F:55.10	259–263	KP278925	2	1.95	0.54	0.49	0.37
		R:CTTTATTGGTAGTGGATTGGAC	R:55.37							
SCF174468	(AG)13	F:CAACATTCTTCGCTCACAA	F:55.81	174–178	KP278926	3	2.52	0.77	0.60	0.54
		R:CTAAGAGTTGACATGATTGGC	R:54.97							

Table S2. *Cont.*

Primer ID	Motif	Primer Sequences (5′-3′)	TM (°C)	Allele Range	GenBank Accession Number	Heterozygosity				
						N_A	N_E	H_O	H_E	PIC
SCF175823	(CT)12	F:AGGGGCAGTTTAGTCCTAGTAT	F:55.38	221–231	KP278927	3	2.25	0.85	0.56	0.47
		R:GCACGTCTTTTCTGTAGTTCAT	R:56.20							
SCF177450	(TC)10	F:TCTAAAACTCTCCTCTCACCTC	F:54.51	268–271	KP278928	3	2.17	0.58	0.54	0.77
		R:GATAGCAGTGGACTCATGTCT	R:54.31							
SCF177451	(AG)10	F:GTACCATATAAGAAAGGGAGCC	F:56.5	183–209	KP278929	8	4.76	0.92	0.79	0.77
		R:CAATAGAAACCCAAGACAACTC	R:55.56							
SCF17979	(TC)17	F:ATATCAGAACAAGGAGATGGTG	F:55.76	210–229	KP278649	6	3.76	0.85	0.73	0.70
		R:GATACCGAATGAACCAAGAA	R:55.13							
SCF180863	(CT)11	F:CCAGTTACAGATCCTTGAGTTG	F:56.49	169–181	KP278930	4	3.35	0.85	0.70	0.65
		R:GCAATGTTCCCTCGAATTA	R:56.16							
SCF181772	(TTA)28	F:AGCAACGTATGGTGGTATC	F:53.36	129–179	KP278931	9	6.38	0.23	0.84	0.60
		R:CATTTGTTTCCACAGCTTC	R:54.16							
SCF181909	(CT)10	F:CTCTCAATCTCTTGTTTTCTCC	F:54.92	172–176	KP278932	3	2.38	0.75	0.58	0.50
		R:TTCAAACCTCAGCAATCAG	R:54.78							
SCF183590	(CT)11	F:TTTGTAGTATGGGGACACTGAT	F:56.11	168–173	KP278933	3	1.89	0.54	0.47	0.42
		R:AAAGAGGCAGGTCAGAAAAT	R:55.60							
SCF18363	(TC)10	F:CAAAGACCGCTAGGTTTACA	F:55.66	170–176	KP278650	3	1.89	0.62	0.47	0.42
		R:ACTGCTCACTAGACAAGATCG	R:54.77							
SCF184873	(TA)11	F:AAGCGTAGAATATGTATGACCC	F:55.9	223–239	KP278934	4	2.94	0.70	0.66	0.61
		R:GGTAGTCCTCACGGAAGAG	R:54.72							
SCF18709	(AC)12	F:GTAATGGTAAGTGTCGAAATCC	F:55.36	342–350	KP278651	4	3.63	0.85	0.73	0.67
		R:CATAGATGTAACCACGCTTCT	R:54.61							
SCF187979	(AG)10	F:AGATAAGGCACCCGATAATAC	F:55.0	201–269	KP278935	6	3.67	0.77	0.73	0.69
		R:GATCAAGGAACGCAAATCT	R:55.26							
SCF189612	(AG)10	F:GAGGATTGTTAATGGTTTCTTT	F:54.25	148–154	KP278936	4	2.89	0.85	0.65	0.59
		R:TACGCTTCATCTTGTTATTTTC	R:54.9							

Table S2. *Cont.*

Primer ID	Motif	Primer Sequences (5′-3′)	TM (°C)	Allele Range	GenBank Accession Number	Heterozygosity				
						N_A	N_E	H_O	H_E	PIC
SCF189657	(AG)10	F:CATCCTTGAAAATAGACAGACC	F:55.48	238–240	KP278937	2	1.80	0.67	0.44	0.35
		R:CTTAGAAGACCGCACTGAGA	R:55.79							
SCF189827	(AG)9	F:TTCATTTCCTTACACTTCCC	F:54.27	184–187	KP278938	3	1.73	0.54	0.42	0.38
		R:GTTAGCTTCTTCTCCTTCTTCA	R:54.86							
SCF191642	(CA)10	F:CTACATCCACTAAATATCAAGGC	F:54.87	228–230	KP278939	2	1.17	0.15	0.14	0.13
		R:GATCAAGCCAAAGGAAGAA	R:55.40							
SCF192074	(TA)9	F:CCTTGGAAAACACCTTTTG	F:55.71	187–189	KP278940	2	2.00	0.54	0.50	0.38
		R:GCCAAACAATATGGGACAG	R:56.39							
SCF192219	(GA)9	F:GAATTTTGTCGTTCCAGAGA	F:55.33	150–158	KP278941	4	2.30	0.62	0.57	0.50
		R:AAAAGAAGAAGAGGAATGGC	R:55.25							
SCF192715	(AG)9	F:CTCTGCCTTGTTCGTCTCT	F:55.60	187–213	KP278942	5	3.45	0.77	0.71	0.67
		R:AACCAATCGAAGGTGACAA	R:56.46							
SCF193103	(GA)11	F:GAGGAGTTGAAACAATTAGTCC	F:54.65	150–168	KP278943	4	2.62	0.69	0.62	0.56
		R:TACCCACTTTAGTCGAAGGAT	R:55.50							
SCF194552	(CT)9	F:CACAGGTGTAGGGTCTTGTT	F:55.5	209–238	KP278944	7	4.23	0.69	0.76	0.73
		R:AAAAGGAGGCAAGGATAGAG	R:55.29							
SCF19565	(CT)13	F:GGGTTTTATGAGTTAGAGTCCC	F:55.95	287–317	KP278652	4	2.62	0.69	0.62	0.56
		R:GTAGGTTTCTTCGATGGTCTT	R:55.6							
SCF197903	(GA)11	F:TCTCGTGAGCGTTACAATATAC	F:54.81	147–155	KP278945	5	2.91	0.77	0.66	0.59
		R:ATGGAGTCAAGGTAAACCG	R:55.5							
SCF199831	(TC)15	F:GTAGGTATCATCGCTGTCTTC	F:54.4	182–201	KP278946	5	3.31	0.77	0.70	0.66
		R:GTGCATCACATACAAGCTCT	R:53.73							
SCF201915	(AC)19	F:ATGCACATCCTGAAGTACCA	F:56.51	188–218	KP278947	8	6.26	0.92	0.84	0.82
		R:CTGAACACATTGGACGGAT	R:56.80							
SCF204332	(TC)9	F:CGTGATCTCCCAGAGTTGT	F:56.3	169–182	KP278948	4	1.77	0.46	0.44	0.40
		R:CTTTTATTTCCCTATGTGTCCC	R:56.72							

Table S2. *Cont.*

Primer ID	Motif	Primer Sequences (5′-3′)	TM (°C)	Allele Range	GenBank Accession Number	Heterozygosity				
						N_A	N_E	H_O	H_E	PIC
SCF204979	(CA)13	F:GGAAAGAGGTAAGAAATGGG	F:55.38	149–153	KP278949	3	1.89	0.62	0.47	0.42
		R:TAAGAGTTCCCACAACCAAA	R:55.72							
SCF20681	(GA)16	F:AGCCTAAACCTCTGTTTGATG	F:56.13	218–233	KP278653	3	1.48	0.39	0.32	0.29
		R:TTACAATACCTCGCTCCTTAGA	R:55.97							
SCF208509 *	(GA)14	F:GCTTCACACTTGATAGTAGGTTG	F:55.82	146–165	KP278950	6	4.83	0.92	0.79	0.76
		R:TACCGCCATTGTAGCAGAT	R:56.75							
SCF208875	(CA)10	F:AAGGAGTTCAGATAGTCAAAGG	F:54.42	172–182	KP278951	2	1.95	0.85	0.49	0.37
		R:AGGAATGAGATGGATATGGA	R:54.35							
SCF208883	(GA)9	F:GAGGAGTGAAGAGCCAGTAA	F:54.63	155–163	KP278952	3	2.00	0.54	0.50	0.41
		R:GACATTTCAAGTCCCACACT	R:54.91							
SCF21119	(TA)9	F:GGATTTGAGGACTATACCAAGA	F:55.2	331–347	KP278654	6	3.84	0.92	0.74	0.70
		R:TTAAAAGGCATACGCTGAC	R:54.10							
SCF213102	(CAG)8	F:GTGAAGATACAGTGGAGAGCA	F:55.46	148–160	KP278953	4	2.25	0.62	0.56	0.51
		R:ATGGTAGTTGTTGACCTGATG	R:54.99							
SCF21596	(AC)11	F:ATATACTGGCATAAACACCCTC	F:54.94	303–320	KP278655	8	5.20	0.92	0.81	0.78
		R:CCTTACTCTTATCATGGCTAGG	R:54.99							
SCF22434	(GA)9	F:TATGTATAGTCCCACAACAAGG	F:54.35	263–265	KP278656	2	1.74	0.46	0.43	0.34
		R:TCCTGTCTATCACTCACATCAC	R:55.12							
SCF22442	(CT)11	F:ACAAAGAAAGACACTCCATCTC	F:55.10	338–348	KP278657	4	2.62	0.77	0.62	0.56
		R:GTATTTGACTTCCATGACCAC	R:54.45							
SCF22477	(CT)12	F:CTCTCCCCTACTTTCTTCCTAT	F:55.3	243–247	KP278658	3	1.77	0.42	0.43	0.37
		R:GCCGCTAACACAATTAACTAAC	R:55.9							
SCF2288	(AGA)8	F:CAATAGTAGTTTCGAGCTTTCC	F:55.17	216–230	KP278600	4	3.22	0.85	0.69	0.64
		R:GTTTCCAATTCAAGCCTCTA	R:54.60							
SCF22962	(AC)9	F:GTGCAACAGCTAACAGCATA	F:55.14	198–204	KP278659	2	1.95	0.54	0.49	0.37
		R:AGGACCAATACTCAGAACAAAC	R:54.99							

Table S2. *Cont.*

Primer ID	Motif	Primer Sequences (5′-3′)	TM (°C)	Allele Range	GenBank Accession Number	Heterozygosity				
						N_A	N_E	H_O	H_E	PIC
SCF23210	(TC)11	F:TTGATACTCTCGACCTCTTCTT	F:54.96	189–209	KP278660	5	4.28	1.00	0.77	0.73
		R:GTGGTGTTCGACATGATTTAC	R:55.42							
SCF23339	(GA)15	F:GCAAAACAGAGTTATAGTGGCT	F:55.49	253–270	KP278661	6	4.51	0.85	0.78	0.75
		R:TAGACAGAAGCACAGATTGGTA	R:55.24							
SCF23691	(TCT)13	F:CGGCTTTGTTAGTTGATGTT	F:55.49	259–281	KP278662	7	5.12	0.92	0.81	0.78
		R:CGATGTTGTACTATTCATGTCC	R:54.71							
SCF24087	(CTT)11	F:GTCCCTTTCTCGTGTCTTTAT	F:55.6	191–212	KP278663	4	2.47	0.54	0.60	0.52
		R:GAGTAGTGACGATGCAACTAGA	R:54.80							
SCF2483	(GTG)8	F:TTTCCTTCATAGTGTTGCCT	F:54.95	169–184	KP278601	2	1.74	0.31	0.43	0.34
		R:GTCTCCCTGTTAAATCCACTC	R:55.29							
SCF25221	(CT)10	F:GTATCCCCACACTTACCACTAT	F:54.69	317–331	KP278664	4	2.75	0.62	0.64	0.66
		R:AGGATTGGACGGTAGCTTA	R:54.89							
SCF25446	(CT)9	F:TAGTGTGGACTTAACATGGAGA	F:54.91	159–161	KP278665	2	1.99	0.62	0.50	0.66
		R:ATCCAACCAAGTATCAGCAA	R:55.67							
SCF259	(CT)10	F:TGACAGTACCAATAGCAGGAC	F:55.33	177–194	KP278591	5	3.49	0.69	0.71	0.66
		R:AACACCCAGTCGTTATACATCT	R:55.30							
SCF25944	(GA)10	F:AACTATGCCAGAAGACTCAGAT	F:54.82	293–321	KP278666	7	4.69	1.00	0.79	0.76
		R:CTTCACAAATCACAACCACTAC	R:54.82							
SCF26014	(GA)9	F:GGTCCCAGAATCAATGTCTA	F:54.94	166–170	KP278667	3	2.89	0.77	0.65	0.58
		R:GAAATCAGAGAAGAAACAGGTC	R:54.75							
SCF26049	(TTC)13	F:GTTCAGGTCTGTTGTAAGGAAG	F:55.63	168–187	KP278668	6	4.28	0.85	0.77	0.73
		R:TTTCTTGTAGGACGAAGTGG	R:55.46							
SCF26697	(CT)13	F:TCGTAACTATTCAGTGGGTGT	F:54.76	272–282	KP278669	5	2.52	0.85	0.60	0.56
		R:GGAGCAGTAGAGATTAAACGAC	R:55.0							
SCF2714	(AG)9	F:ACAAGTCTCTGGAAGCTAACAT	F:54.90	202–208	KP278602	4	2.60	0.85	0.62	0.54
		R:GTTGATTGTTGGGTCTAAGTTC	R:55.39							

Table S2. *Cont.*

Primer ID	Motif	Primer Sequences (5′-3′)	TM (°C)	Allele Range	GenBank Accession Number	Heterozygosity				
						N_A	N_E	H_O	H_E	PIC
SCF27510	(GA)13	F:CCTTCAGATTCAACGTATTCTC	F:55.63	239–284	KP278670	8	5.73	1.00	0.83	0.80
		R:GGTGTATCACATCCCAAAAC	R:55.15							
SCF27755	(GA)11	F:GAAGTGAGAGTAGGAATCGAAG	F:54.96	335–357	KP278671	6	3.98	0.92	0.75	0.71
		R:CCACAACACAAAACCCTAAT	R:55.0							
SCF27811 *	(TC)10	F:ATGTGACTAGCATGGGACTTA	F:54.84	219–259	KP278672	4	2.97	0.77	0.66	0.61
		R:TATTTACCTGGATAGGAGAAGG	R:54.38							
SCF27934	(AC)9	F:TCCAAATAGCCCAGAATAAG	F:54.60	234–237	KP278673	2	2.00	1.00	0.50	0.38
		R:GGTACTCCCATGTAATTGTTGT	R:55.55							
SCF28100	(TCT)7	F:TAGAAACTAACATGGGAGGTGT	F:55.43	239–295	KP278674	6	5.93	1.00	0.83	0.81
		R:GCACGCTGTATTGATAGAAGAT	R:56.23							
SCF28279	(TC)12	F:GATACTTTACCTCCTCCTCAAG	F:53.98	231–239	KP278675	4	1.63	0.46	0.39	0.36
		R:TTGTCCTCTATCTCTAACTCCC	R:54.34							
SCF28509	(GA)9	F:GCAAACACCACACTATATGAGA	F:55.35	212–221	KP278676	5	2.86	0.77	0.65	0.59
		R:ATAGAGAACCACAGAACAGGAC	R:54.97							
SCF28613	(CAA)16	F:CATTCTTCACTCCAACTTCAG	F:55.1	182–220	KP278677	5	2.94	0.92	0.66	0.60
		R:CAAGTCCCATCATCATTTTC	R:55.38							
SCF28931	(TC)13	F:TCTCATAAGTCAGAACCTCACA	F:55.2	225–229	KP278678	5	4.07	0.69	0.75	0.71
		R:CTAAACTAAACCTCCTAACCGA	R:54.68							
SCF28955	(GT)9	F:TATTCAAAGCCACTAGGCAC	F:55.57	235–246	KP278679	5	3.45	0.89	0.71	0.67
		R:CAAACCAAATTCTCCTTCTG	R:54.93							
SCF29560	(GA)13	F:GTGTGGTGTGGTCTCTACAAT	F:54.87	248–258	KP278680	5	2.41	0.62	0.59	0.53
		R:ACATCTCTTTGGCTGATACTTC	R:55.22							
SCF29735	(TG)16	F:CGTAAAATCTGTTGTCTCTGTG	F:55.16	263–278	KP278681	5	3.84	0.85	0.74	0.70
		R:TCTCTATGCTCCTTCCACTTAT	R:54.93							
SCF30000	(AGA)10	F:GACTCTTCAACTTCCACGTTA	F:54.59	161–167	KP278682	3	1.98	0.69	0.49	0.43
		R:GAAATCTTAATCTTGCAGCC	R:54.28							

Table S2. *Cont.*

Primer ID	Motif	Primer Sequences (5′-3′)	TM (°C)	Allele Range	GenBank Accession Number	Heterozygosity				
						N_A	N_E	H_O	H_E	PIC
SCF30010	(CT)9	F:CTCAAATCAACGATCAAGAC	F:53.16	302–337	KP278683	8	4.33	0.92	0.77	0.74
		R:GAAAGAGACAACAAAACCCT	R:53.45							
SCF30734	(TTA)8	F:GTTGAAAACCCAACTGTGAG	F:55.63	178–240	KP278684	6	2.89	0.69	0.65	0.62
		R:AGATCCAGTCATGGTACTTTTG	R:55.83							
SCF30816 *	(TCG)8	F:GTCCAAAATAGCATCGAAAG	F:55.5	207–216	KP278685	3	1.89	0.62	0.47	0.42
		R:CGCATTACTTCTTCACTATACG	R:54.60							
SCF31172	(CT)11	F:ACTGGATCTGGTGTTATTTACC	F:54.81	161–167	KP278686	3	2.70	0.77	0.63	0.56
		R:GGCTGGAAACAATTCAAAC	R:55.61							
SCF31208	(CT)10	F:AACAGCACCACTACAACACTT	F:54.89	306–359	KP278687	5	3.60	0.85	0.72	0.68
		R:AGAGAACAATCGTCTAATCGTC	R:55.22							
SCF31394	(TC)11	F:GTAGCAAAAGAAGAGACACCAT	F:55.30	273–287	KP278688	5	3.80	0.85	0.74	0.69
		R:CGTTTTCCAGTTCCAGAGTA	R:55.46							
SCF3191	(TCT)15	F:GCACTATCAGGAAGAGGAATTA	F:55.32	238–260	KP278603	5	3.71	0.92	0.73	0.69
		R:GTAACACCAGAAAACAACTGC	R:54.96							
SCF3261	(TC)10	F:GTTTACCATATTCACTCCTTCC	F:54.48	260–268	KP278604	3	2.77	0.77	0.64	0.57
		R:TGAGACAGACCTAACATTTGAC	R:54.43							
SCF32727	(TC)18	F:ATGTAACGGTCTCCACTTTCT	F:55.41	192–210	KP278689	8	5.45	0.92	0.82	0.79
		R:TAGTATCTTCGTGGTCAGAGGT	R:55.59							
SCF33047	(GCT)8	F:TAGGGAAGGAGTAGTTATCGAA	F:54.96	161–173	KP278690	4	2.44	0.82	0.59	0.52
		R:ATGCTGACCTCATCGTCTT	R:55.65							
SCF33185	(CT)14	F:AGCACACTACAGACAGGGTAAT	F:55.53	198–202	KP278691	3	2.62	0.69	0.62	0.55
		R:GTTTTGGCTCTGGCTAAGTAT	R:55.41							
SCF33471	(TC)12	F:TTTATTGCACACGAGAACAG	F:54.94	288–329	KP278692	7	2.70	0.46	0.63	0.61
		R:ATATTTTGTCCACGCTCACT	R:54.81							
SCF3362	(TGC)9	F:GTACAGCAAAATTCAGCACA	F:54.93	343–372	KP278605	3	2.25	0.46	0.56	0.47
		R:GGATTTATCTACAGCCCATTAC	R:54.62							

Table S2. *Cont.*

Primer ID	Motif	Primer Sequences (5′-3′)	TM (°C)	Allele Range	GenBank Accession Number	Heterozygosity				
						N_A	N_E	H_O	H_E	PIC
SCF34010	(CA)10	F:GAGAATATGTGATGTTGAGGTG	F:54.64	285–287	KP278693	2	1.35	0.31	0.26	0.23
		R:CAAGTGTTAGGCTCGTTTAGTT	R:55.57							
SCF34071	(TG)10	F:CGTGTGCAGATTTACTTCAG	F:54.50	268–276	KP278694	3	2.94	0.85	0.66	0.59
		R:AATTCATAGATCCCCATGAC	R:53.76							
SCF3427	(CT)11	F:GCAAGACATCATCACAAACA	F:55.46	347–349	KP278606	2	1.35	0.31	0.26	0.23
		R:CTTATCCCAGTCCTTCAACTTA	R:55.26							
SCF34513	(TG)10	F:TACTAATCTTCTGGTTTGGGC	F:56.5	228–232	KP278695	2	1.47	0.40	0.32	0.27
		R:GTACACCACTCCTGATGGC	R:56.34							
SCF34584	(AG)10	F:GTCTGTTTGGAAGAAGAAGGT	F:55.9	199–213	KP278696	4	3.49	0.85	0.71	0.66
		R:CTGTTCGTCAATCCCTAGC	R:55.82							
SCF35507	(GA)9	F:GTCTAATCTAATGCAGAATGCC	F:55.73	238–240	KP278697	2	1.55	0.46	0.36	0.76
		R:AATGTGGACAACGAGTACATCT	R:56.19							
SCF3551	(AC)9	F:CTTCGACGTTTCTGTGACTAT	F:54.63	274–289	KP278608	6	4.76	0.92	0.79	0.76
		R:AGTTGGTGATTGGAAGAGTAAG	R:55.16							
SCF3595	(CA)10	F:AGACTACAGTGAACAAAGACCA	F:54.9	316–332	KP278607	6	4.90	1.00	0.80	0.77
		R:CTGACTTGGTGTGATTAGTGAG	R:54.97							
SCF36745	(TC)14	F:TCCTCATTAAGTATTGGACAGG	F:55.92	307–316	KP278698	4	2.94	0.69	0.66	0.60
		R:CTGGATTCTTGTTCTTAGCTTC	R:55.13							
SCF37023	(AAT)12	F:GAATAGCCTTAACATACGCTGT	F:55.46	332–351	KP278699	3	2.74	0.67	0.64	0.56
		R:ATTGGAATGGTTTAGTGGTG	R:54.92							
SCF37628	(GA)12	F:ACCAGCTCAGATAACAATGC	F:55.39	255–263	KP278700	2	1.90	0.62	0.47	0.36
		R:GAGTAGGATACCTCCACACCTA	R:54.96							
SCF38340	(TC)9	F:CAAACCATTTTAACGGAGAG	F:54.99	336–339	KP278701	3	1.27	0.23	0.21	0.20
		R:AATCATCGTGCATACCTGTT	R:55.52							
SCF38430	(GA)14	F:CAATAGTTAGGAAGTTGGAACC	F:54.73	156–176	KP278702	3	1.91	0.62	0.48	0.39
		R:CTAAGAACCAAACAGAGCCTTA	R:55.63							

Table S2. *Cont.*

Primer ID	Motif	Primer Sequences (5′-3′)	TM (°C)	Allele Range	GenBank Accession Number	Heterozygosity				
						N_A	N_E	H_O	H_E	PIC
SCF38553	(GA)10	F:CTTCTGTTTACTCACTTCCACC	F:55.63	350–358	KP278703	4	2.09	0.46	0.52	0.48
		R:ATGGTCCCAAGATACTTTAGC	R:55.3							
SCF38942	(CT)11	F:CTTGCTATTTGGTACTCGTCTT	F:55.50	230–240	KP278704	3	2.86	0.69	0.65	0.58
		R:CTTGACAGTTATTTCTCTTCGG	R:55.87							
SCF3914	(TAC)13	F:TGTGGAGTTAGAGTGACATACC	F:54.30	345–372	KP278609	5	3.98	0.85	0.75	0.71
		R:GACAAGAATGATGAGTAGCGT	R:54.52							
SCF39242	(TC)14	F:ACTCCTGAAGAAGAAGAACAGA	F:54.98	246–276	KP278705	7	4.76	1.00	0.79	0.76
		R:AATGAATGCAGACCACAGAT	R:55.48							
SCF3932 *	(TC)10	F:CAGAGTTTCAGTGGAGCATT	F:55.44	311–319	KP278610	5	3.31	0.77	0.70	0.66
		R:CTCAGCTTCTGTGTTTTGTGT	R:55.62							
SCF39705	(AT)13	F:GCAGGTAAATCCTATCTGGAAT	F:56.43	333–397	KP278706	7	3.08	0.70	0.68	0.64
		R:GTTGAAGACACCTAGTCCACTC	R:55.46							
SCF40517	(CT)12	F:GTAGAATGGCAATAGGGTTT	F:53.47	245–259	KP278707	5	3.05	0.92	0.67	0.62
		R:GAAGAAGATGACGAAGATCAC	R:53.91							
SCF41361	(CA)9	F:AAAATTGCTTGGTCCTCAC	F:55.17	231–233	KP278708	3	1.61	0.31	0.38	0.34
		R:AAGTGTATAGTCTGGGGTGTTC	R:54.87							
SCF41971	(GA)12	F:ATACTTGACCTCTATGGCTTGA	F:55.67	280–300	KP278709	7	4.83	0.92	0.79	0.77
		R:GTACTTACGTGTTTGGTTCGTT	R:55.99							
SCF42332	(CT)10	F:GATAGAATGACGAAACTAACCC	F:54.64	194–208	KP278710	4	2.60	0.92	0.62	0.54
		R:AGTGGGGAGATAATTGAGAAG	R:54.99							
SCF4305	(GA)13	F:AATGAGTGGTTATGTAGGGAGA	F:55.36	179–191	KP278611	5	4.76	0.85	0.79	0.76
		R:AGATTGGTGAGATATGAGGAAG	R:55.0							
SCF43145	(TCT)7	F:TGGTTTTGGATACACACTTG	F:54.42	235–261	KP278711	7	6.76	0.69	0.85	0.83
		R:AAGAACAAGATCACCACTCTG	R:54.38							
SCF43220	(TC)14	F:CTTGTCGAGCATCCTATATTTC	F:55.74	154–159	KP278712	4	2.50	0.69	0.60	0.52
		R:AAAAGTCATGGGAAGGTGTT	R:56.6							

Table S2. *Cont.*

Primer ID	Motif	Primer Sequences (5′-3′)	TM (°C)	Allele Range	GenBank Accession Number	Heterozygosity				
						N_A	N_E	H_O	H_E	PIC
SCF4386	(TTC)9	F:GTTACTCATTTCTTTGCTGAGG	F:55.86	200–203	KP278612	2	1.99	0.62	0.50	0.37
		R:CCTCTTAGTGTTGGAGTTTCAT	R:55.16							
SCF45712	(CA)9	F:GCAGTGTGCTTTTCTTTTCT	F:54.90	206–210	KP278713	2	1.35	0.31	0.26	0.23
		R:GTTACTAGGGTACTGGGTTTGA	R:55.5							
SCF46588	(TG)11	F:ACAAACCTTGAGCCTATTTG	F:54.56	350–356	KP278714	5	2.06	0.46	0.52	0.47
		R:GTCTGAGTTTCCACTATCGTCT	R:55.13							
SCF46739	(TC)10	F:ATGTTAGGTGATGCTGTTGTC	F:55.15	247–249	KP278716	2	1.83	0.69	0.45	0.35
		R:CAGGTGCTTATTTTCGTTTC	R:55.13							
SCF46751	(AG)12	F:ACCAGATGAAGAAGAAGAAGC	F:55.33	309–325	KP278715	5	3.07	0.85	0.68	0.63
		R:GCCTCTCATTACCATTACAAAC	R:55.51							
SCF46824	(ATT)10	F:GGAGATGCTGTAATAACGAAGT	F:55.27	193–209	KP278717	5	3.80	0.77	0.74	0.70
		R:TTAGTCAATATGCGTGCAAC	R:54.90							
SCF46833	(AAC)7	F:GGACCGCCGTATTTAGTTA	F:55.36	208–211	KP278718	2	1.74	0.46	0.43	0.34
		R:GCCCATACCCCTAGTTATTG	R:56.12							
SCF46912	(AG)9	F:GAACAATAAAGAGGCTAGAGGA	F:54.68	172–182	KP278719	4	2.50	0.69	0.60	0.55
		R:CATAGTTGTAGAGAAGATCGGG	R:55.68							
SCF47809	(CAT)10	F:CTTCTACCTTCCAAGATTTGTG	F:55.72	289–304	KP278720	2	2.00	1.00	0.50	0.38
		R:ATTACTATTCCCAGAGACGACC	R:56.38							
SCF48414	(GA)12	F:GTAGGGAAACAAGAATTGGAC	F:55.30	284–286	KP278721	2	1.95	0.69	0.49	0.37
		R:ACTGTGAGATTGGTGTGATATG	R:55.1							
SCF48645	(CT)10	F:AAAATAGGTCCCACATGAGTAG	F:54.98	310–312	KP278722	3	1.37	0.31	0.27	0.25
		R:GCTAGACGATGACACATTATTC	R:54.10							
SCF49598	(TCT)8	F:ATGAGGTTTTCCAACACAAC	F:54.93	258–261	KP278723	2	1.83	0.69	0.45	0.35
		R:TCAGAGGGAAGTACATGAGAAT	R:55.48							
SCF49656	(TC)14	F:ACTCTTACCCTTGAAACCAACT	F:56.10	284–301	KP278724	5	3.71	0.85	0.73	0.69
		R:TAGGTGCATGAGACTTTTAACC	R:56.13							

Table S2. *Cont.*

Primer ID	Motif	Primer Sequences (5′-3′)	TM (°C)	Allele Range	GenBank Accession Number	Heterozygosity				
						N_A	N_E	H_O	H_E	PIC
SCF51810	(TGC)8	F:TATTACTCTGTTGCTGCTGTTG	F:55.91	189–204	KP278725	3	2.18	0.69	0.54	0.46
		R:ACTAAACCCTAATGTCCCTTCT	R:55.21							
SCF53282	(GA)11	F:GACAATCACATACCCATAACAG	F:54.55	214–234	KP278726	3	1.61	0.46	0.38	0.34
		R:CCACTCTTTCCCTCTATCG	R:54.85							
SCF53750	(CA)10	F:GTTTCATAGAGATGGGTTTCTG	F:55.48	316–353	KP278727	6	2.41	0.69	0.59	0.56
		R:CTTGGTTCCCTAAGCTACATT	R:55.27							
SCF54155	(GA)14	F:TCGAAGAAAATGAAGGGAC	F:55.24	292–335	KP278728	8	5.54	0.77	0.82	0.80
		R:ACAAATGGAGAGGAAAGTGTAG	R:55.16							
SCF55511	(GA)9	F:GAAGTGAAAATCTGAACCTCTC	F:54.75	178–183	KP278729	2	2.00	1.00	0.50	0.38
		R:ACTCTCGAATCTGTCTTCTTGT	R:54.84							
SCF55751	(CT)10	F:ACTCACGTCCATTTTCTCAC	F:55.9	227–239	KP278730	6	4.97	0.62	0.80	0.77
		R:AGCGATATAACAATACCAGAGC	R:55.38							
SCF56032	(GAT)11	F:AGAAATGGCGCTCTGTATC	F:55.46	195–216	KP278731	3	2.25	0.85	0.56	0.47
		R:GAACAGTCTCATCTTCACGAC	R:54.78							
SCF56561	(AG)13	F:ATTAGCCATTCGTGATTAGG	F:54.46	218–230	KP278732	4	2.84	1.00	0.65	0.58
		R:TAAGGAGATACGACCAAGAAAC	R:55.25							
SCF56717	(AGG)7	F:GTGTTTGTGTTTGTGTCTGTG	F:54.91	199–211	KP278733	2	1.99	0.77	0.50	0.37
		R:GATGATTTCACCTACATCGG	R:55.42							
SCF56747	(GA)14	F:TTAGAGAAAGGTCCCAACAG	F:54.50	256–280	KP278734	7	5.54	1.00	0.82	0.80
		R:GAAGAGGCTAAGAGGTCATGT	R:55.20							
SCF56816	(AGC)9	F:CGGATTGACTAATTTCTGTCTC	F:55.63	210–219	KP278735	2	1.55	0.46	0.36	0.29
		R:CTCTTATTCCACCAAACGAA	R:55.39							
SCF57479	(CT)10	F:AAGTGCAAGTGTGAGAGTGTAT	F:54.11	196–204	KP278736	4	2.50	0.62	0.60	0.52
		R:TGATGGGTGTAAGTGTAAAGAG	R:54.52							
SCF57497	(TC)10	F:ATCTGTAGGTTGTGTTACCCC	F:55.53	239–240	KP278737	2	1.90	0.77	0.47	0.36
		R:ATCAACTGTATCTACCCACCAA	R:56.11							

Table S2. *Cont.*

Primer ID	Motif	Primer Sequences (5′-3′)	TM (°C)	Allele Range	GenBank Accession Number	Heterozygosity				
						N_A	N_E	H_O	H_E	PIC
SCF58861	(TA)10	F:GTTGACTAAAAGGCATTGGA<br>R:GACTACTATTTTCTGCACAGGG	F:55.38<br>R:55.74	147–164	KP278738	7	6.15	1.00	0.84	0.82
SCF59035	(TC)16	F:AGATTTTGAACGATGTCTGC<br>R:GATCTATCGCTTATCCAGTACG	F:55.36<br>R:55.77	301–336	KP278739	5	3.28	0.92	0.70	0.65
SCF59248	(TTA)7	F:TAGTTGAAAATGGAGAGAGAGC<br>R:TTAGATGCCCAACACTACATC	F:55.51<br>R:55.26	194–204	KP278740	4	2.94	0.77	0.66	0.61
SCF59739	(CT)11	F:GTATGACTGTACCAAACAAACC<br>R:CAGCTTTCCCTTCTAAATGA	F:53.79<br>R:54.78	334–350	KP278741	5	3.45	0.77	0.71	0.66
SCF60761	(GA)10	F:ACTTAAACATCGGTCCATAGAG<br>R:AGAGTCGTGTCCTTTCTTTTC	F:55.13<br>R:55.25	259–263	KP278742	3	2.12	0.55	0.53	0.47
SCF61078	(GAA)7	F:GACTCTTCATATAACCCACAGC<br>R:AAAAGTGCTTGATCGTTAGC	F:55.50<br>R:54.87	252–254	KP278743	2	1.17	0.15	0.14	0.13
SCF61189	(AG)9	F:GCCATAACTCTCACTCAAATCT<br>R:ACCTATTCACCTACATCCAAAG	F:55.22<br>R:54.98	307–319	KP278744	5	3.49	0.69	0.71	0.66
SCF6195	(AG)14	F:GACTATGAATCTGACGCTCAC<br>R:CCAGTAAATACGTGACTAATCG	F:54.93<br>R:54.30	340–354	KP278613	5	3.52	0.85	0.72	0.67
SCF64185	(TG)9	F:CACCTCATTTGGTTCATTCT<br>R:CAGATACTAAAGGTTGCCGTA	F:55.3<br>R:54.81	316–320	KP278745	3	2.43	0.77	0.59	0.50
SCF64632	(CT)12	F:ACCTCCTAAAACACAACCCTA<br>R:CTGAGTAATCTTCGATGTGAGA	F:55.42<br>R:54.69	149–175	KP278746	7	4.12	0.85	0.76	0.72
SCF64758	(TG)11	F:TAAGAGGGTTTGAGCATTCA<br>R:TTGGGTCATAAACAACCTCA	F:55.97<br>R:56.45	350–356	KP278747	3	1.90	0.46	0.47	0.43
SCF6530	(CT)9	F:CCCCAAGTATAATGTGTAAAGG<br>R:AGTTCGCATAGAAACTGTAGGA	F:55.41<br>R:55.89	349–353	KP278614	2	1.95	0.54	0.49	0.37
SCF65999	(CT)11	F:AGGTAGCATTAGACACGAGATT<br>R:GAGGTTTTACATGACCATTACC	F:54.88<br>R:55.20	288–300	KP278748	2	1.74	0.46	0.43	0.34

Table S2. *Cont.*

Primer ID	Motif	Primer Sequences (5′-3′)	TM (°C)	Allele Range	GenBank Accession Number	Heterozygosity				
						N_A	N_E	H_O	H_E	PIC
SCF66692	(CT)10	F:AAAGTGTATTGGACGGCTG	F:56.20	245–259	KP278749	7	4.02	0.92	0.75	0.72
		R:TTGTTATGGCCCCTCATTA	R:56.46							
SCF68870	(AG)11	F:GTGAATTGTTGCAGAGTACCTA	F:54.67	166–168	KP278750	2	1.08	0.08	0.07	0.07
		R:TGAGTTGAGTTCATATAGCTGG	R:54.76							
SCF6926	(CT)10	F:ACATGCACTTCAAATAGTACCC	F:55.85	212–234	KP278615	4	2.09	0.46	0.52	0.48
		R:TTACAACTTACACAGGAAGCAG	R:54.92							
SCF69698	(TC)9	F:GAGGAGATAAAGGTTTGTTGAG	F:54.82	297–303	KP278751	3	1.81	0.46	0.45	0.38
		R:CTTTGAGACTTTGAGTGAGACA	R:54.80							
SCF69981	(CT)9	F:AGCGTTACCACCGAATATAA	F:54.98	226–244	KP278752	8	6.38	0.92	0.84	0.82
		R:CGAGATATAGTTAAAAGGACGG	R:55.1							
SCF71136	(CAA)7	F:TCTGTTTTCACAGCTATCACAC	F:55.55	179–182	KP278753	2	1.26	0.23	0.20	0.18
		R:GTTCATCAAAGGCCAGAGT	R:55.16							
SCF71184	(ATT)22	F:TCTGTTCAGTTGGGCTTTAT	F:54.95	200–231	KP278754	7	4.97	0.92	0.80	0.77
		R:GCTCACATTCACCTGTAATTC	R:54.79							
SCF7132	(TC)10	F:AAGGGGAAGGACAATAAGAA	F:55.38	218–236	KP278616	3	1.98	0.54	0.49	0.43
		R:AATTTGATGACTGTTGTGGC	R:56.1							
SCF7155	(GT)11	F:GGGATCTATGAGTTGTGGACTA	F:55.76	168–196	KP278617	4	1.91	0.54	0.48	0.43
		R:CCACGGAATAGTTGTAAGTTGT	R:55.93							
SCF72209	(CT)12	F:CTTTACCTTTTCCTTCAGTCGT	F:56.77	206–210	KP278755	3	2.94	0.92	0.66	0.59
		R:GAGGTTCACCAAATCTTACCA	R:56.65							
SCF72229	(CCA)7	F:CAACTTCTACAACCACTCCAC	F:54.78	322–334	KP278756	3	2.27	0.60	0.56	0.50
		R:GATTTATTGTGCTACACTGGTC	R:54.3							
SCF72379	(GA)12	F:TAAGGAGATCGACTAGGGTTT	F:54.76	202–217	KP278757	5	2.36	0.62	0.58	0.54
		R:CATCAAGATTCAAGACCACAC	R:55.56							
SCF73288	(TC)12	F:CAGAGGAACAGCAGACTACAT	F:54.59	226–334	KP278758	5	3.89	0.77	0.74	0.70
		R:CCTAGTACGTCATTGGACATTA	R:54.50							

Table S2. *Cont.*

Primer ID	Motif	Primer Sequences (5′-3′)	TM (°C)	Allele Range	GenBank Accession Number	Heterozygosity				
						N_A	N_E	H_O	H_E	PIC
SCF7357	(CT)9	F:CAGCTTAATCATCAGTTCCAG	F:55.14	282–286	KP278618	2	1.65	0.39	0.39	0.32
		R:AGTGAGCATCGACTATTTACCT	R:54.88							
SCF74458	(CT)10	F:GCAGGAAGCTATGATTAAGGTA	F:55.51	222–240	KP278759	4	2.43	0.62	0.59	0.53
		R:TTGAATAGTGTCAGTGGAGAAG	R:54.62							
SCF74895	(TC)9	F:GTACTCCTCTCCGTCTAGCAT	F:55.24	153–219	KP278760	9	6.76	1.00	0.85	0.84
		R:GATTTTATGCGTTAGCTCCA	R:55.65							
SCF75572	(TGA)7	F:GACAAGTGGTTGGGGATAC	F:55.12	247–265	KP278761	3	2.05	0.46	0.51	0.46
		R:ACCCTCATCATCACTCCTT	R:54.24							
SCF7569	(AC)10	F:CCCAATAACGACTCATATACCT	F:54.95	279–283	KP278619	3	2.25	0.62	0.56	0.47
		R:ACCCAGTCAAAATCTCCTTT	R:55.28							
SCF76310	(TC)11	F:CTGTGTAGAACTGCATCAAAAC	F:55.14	220–226	KP278762	4	2.50	0.77	0.60	0.55
		R:TCCTAGAGACCAACCCAATAC	R:55.75							
SCF77145 *	(TG)10	F:TAGAATTAGCCTCCAAGAAGTG	F:55.56	314–325	KP278763	3	1.86	0.54	0.46	0.40
		R:AGAACTAGAAACACGAGAACGA	R:55.94							
SCF77376	(AAG)11	F:CTCATCAAAAGAGAGGAGAACT	F:54.51	256–273	KP278764	6	4.33	0.85	0.77	0.74
		R:TGTAACCAATCTTCATGCTG	R:54.68							
SCF77645 *	(TC)10	F:GGTTCTTTCTTCTGGGTTTT	F:55.1	298–343	KP278765	7	3.25	0.85	0.69	0.66
		R:TCAGACAATGAGCTACTACCCT	R:55.74							
SCF78184	(CA)10	F:CACATTTAAGAGCTACCACCTT	F:55.35	260–276	KP278766	5	3.76	0.85	0.73	0.69
		R:GGTGAAAGAGAAGACTGGATT	R:55.0							
SCF7845	(CT)12	F:GTTCTGACTATTGTGATGGGTT	F:55.67	254–256	KP278620	2	1.90	0.62	0.47	0.36
		R:TGCAATGAATACTGGAAGTG	R:54.68							
SCF79014	(CT)10	F:TCTCTGTCTCTGTCTCTGTCTG	F:54.75	181–183	KP278767	2	1.94	0.46	0.48	0.37
		R:CCAAATCAAGGTCTGTCTATCT	R:55.8							
SCF79620	(GA)15	F:TAATAGCCCTTATACCTGCACT	F:55.3	187–218	KP278768	8	4.97	0.85	0.80	0.77
		R:GAGCATAGACAGCATACAAAAG	R:54.53							

Table S2. *Cont.*

Primer ID	Motif	Primer Sequences (5′-3′)	TM (°C)	Allele Range	GenBank Accession Number	Heterozygosity				
						N_A	N_E	H_O	H_E	PIC
SCF804	(GA)15	F:CAGTCAACAGAGAATACACCAC	F:54.79	221–231	KP278592	4	2.60	0.77	0.62	0.54
		R:TTCCCTATGAAAATCCACAC	R:54.96							
SCF80520	(TC)9	F:TAAAGTGTTTTGGACGGCT	F:55.9	172–178	KP278769	3	2.30	0.62	0.57	0.47
		R:GCACAAATTATCGGAATCG	R:56.62							
SCF80703	(AC)17	F:GGTCTTTCTCCTAATCTCCAA	F:55.11	196–220	KP278770	9	4.69	1.00	0.79	0.77
		R:GGAACCCCTAAATAACATACAG	R:54.56							
SCF81294	(CT)9	F:CTATCGACGGCTGAGATTT	F:55.48	232–238	KP278771	3	2.07	0.69	0.52	0.42
		R:AAAAGGGGAAGATCCTAGAAG	R:55.77							
SCF8151	(CTA)7	F:CGTGCTAGAAGACGAGGTAT	F:54.73	311–314	KP278621	2	2.00	1.00	0.50	0.38
		R:TTAGGGAACAGTAGAAAGGAAG	R:54.62							
SCF81732	(AG)9	F:CGAGTATGTGGAGAGGCTTAC	F:56.58	295–315	KP278772	3	1.89	0.62	0.47	0.42
		R:GTGTATAAAATGGGCATCACAC	R:56.92							
SCF81909	(GT)12	F:TAGAGGAATCAGCAACTTCACT	F:55.87	311–322	KP278773	4	2.58	0.54	0.61	0.55
		R:TTCACACTCACACTCACACG	R:56.57							
SCF8223	(AG)11	F:CATTTAGCATCCATCCATTC	F:55.51	341–355	KP278622	5	2.79	0.69	0.64	0.59
		R:GACTGTGGGTTATTCCTTGTAT	R:54.81							
SCF82535	(AG)9	F:TAGAAGAGGAAAACTGACGGA	F:56.68	245–253	KP278774	4	2.52	0.69	0.60	0.56
		R:TTGATGCAATCTGACAACG	R:56.61							
SCF82870	(CTT)8	F:GCTAAAGAACGAACAACAACAC	F:56.66	261–314	KP278775	7	4.51	0.92	0.78	0.75
		R:GTCCAACGAGTGAGTAGAGAAG	R:55.79							
SCF83036	(TC)14	F:CAACAGTCCTCAAAATCACTC	F:54.82	313–323	KP278776	5	4.02	0.85	0.75	0.71
		R:GTGAACAGAAGTAGAGATCGG	R:54.6							
SCF83615	(TTC)11	F:ATTAGTCGATCTCCTTTTCCTC	F:55.77	323–345	KP278777	5	3.76	0.62	0.73	0.69
		R:AAATTGTAGAGCCAACACTAGG	R:55.35							
SCF83971	(TTG)8	F:ATTCTGGTACTGTTTGTTGCTC	F:56.5	270–347	KP278778	2	2.00	1.00	0.50	0.38
		R:GTTATGTTTCGTGTTCCACTCT	R:55.90							

Table S2. *Cont.*

Primer ID	Motif	Primer Sequences (5′-3′)	TM (°C)	Allele Range	GenBank Accession Number	Heterozygosity				
						N_A	N_E	H_O	H_E	PIC
SCF84804	(CA)13	F:CTAGTCTTCTTGTGACCTAGCC	F:55.7	207–213	KP278779	3	1.62	0.46	0.38	0.35
		R:TATTCTTTTAGTCCGAGCCA	R:55.22							
SCF85773	(GA)12	F:TCTTGAACACAGCACAACAT	F:55.8	281–301	KP278780	8	6.26	0.77	0.84	0.82
		R:ATAAGTTTGCCCCTTTTGTC	R:55.88							
SCF85946	(TC)10	F:TGTGAACAGAACCTACCACTAA	F:54.99	324–328	KP278781	3	2.20	0.62	0.54	0.48
		R:AAAGAGCCCCGTAGATAGAT	R:54.69							
SCF86438	(ATT)7	F:CTATTGAAAACAAGGAACGG	F:54.99	336–340	KP278782	2	1.65	0.54	0.39	0.32
		R:CCTATACAACCTCTTCGGATAA	R:55.23							
SCF87990	(TC)10	F:GTGTAGGTGTAAATGTGCTTTG	F:55.3	260–262	KP278783	2	2.00	0.54	0.50	0.38
		R:GGCGTATAAAAGGATTCAAG	R:54.21							
SCF88396	(GA)9	F:ATAGAGGTTAATTGGTCCTCG	F:55.4	284–292	KP278784	3	2.30	0.62	0.57	0.47
		R:GACGAAGAACGACAGGTAGAT	R:55.98							
SCF8850	(GA)10	F:GTGTGATGTATTTAAGGAGTACCAC	F:56.	344–356	KP278623	6	5.37	0.92	0.81	0.79
		R:ACAGATAGAGTAGTTACCAAGGGA	R:55.45							
SCF88902	(TC)9	F:GTGTTGTAGGATGAACCGAT	F:54.88	315–368	KP278785	5	4.75	0.91	0.79	0.76
		R:GATTTCCAGCATTTGATCTC	R:54.73							
SCF89247	(GA)11	F:TGGAGGAGGTGAAGAATACTAA	F:55.64	198–218	KP278786	6	4.57	0.85	0.78	0.75
		R:CCCTTTGGACAACAAAATAC	R:54.65							
SCF89447	(TC)9	F:TAAATAAGACCTTCTGCTGACC	F:55.40	192–196	KP278787	2	1.45	0.39	0.31	0.26
		R:AATATGCTCACCACCAGTAAAG	R:56.1							
SCF89672	(GA)14	F:CCACTATAATCTACCCCAAAGA	F:55.8	194–200	KP278788	3	2.43	0.31	0.59	0.50
		R:TACTACTGCCCCATCCTACTAC	R:55.16							
SCF89726	(TC)9	F:TTGCTGACTTGCTAACCCT	F:56.7	327–329	KP278789	2	1.65	0.23	0.39	0.32
		R:ATTTACCGAACGCTACGAGT	R:56.57							
SCF89801	(CT)14	F:TAAACCTGTTCCGTCTCTTAGT	F:54.93	323–338	KP278790	7	6.48	0.56	0.85	0.68
		R:CTTTACTGTTGTGTTGTCTGCT	R:54.81							

Table S2. *Cont.*

Primer ID	Motif	Primer Sequences (5′-3′)	TM (°C)	Allele Range	GenBank Accession Number	Heterozygosity				
						N_A	N_E	H_O	H_E	PIC
SCF8987	(CT)11	F:AATCTTTGTCTGAGGTAAGTGG	F:55.16	150–156	KP278625	4	3.71	0.85	0.73	0.68
		R:AACCAGTGTAGTGCAGTTTATG	R:54.63							
SCF90229	(AG)10	F:GTACTTTTGTGGAACTTAACGC	F:55.80	280–292	KP278791	5	2.79	0.69	0.64	0.59
		R:CTGTCCTTTCACTCCTCTTTT	R:55.26							
SCF9068	(TC)14	F:AAATCTAGGTAGGAGCAGGTCT	F:55.49	174–186	KP278624	6	3.89	0.92	0.74	0.71
		R:ATGGAGGAGGAGATATGTGAT	R:55.0							
SCF915	(GA)9	F:TTAGGGTTTGGAGTACCTGA	F:54.80	265–269	KP278593	3	2.54	0.77	0.61	0.68
		R:ACTACCGTCTTTCTTTATAGCC	R:53.86							
SCF9157	(GA)9	F:GGCTTAACAAATTAGCCCTT	F:55.33	301–330	KP278626	5	3.49	0.85	0.71	0.68
		R:GAGAGGATTTACCGACAAAGTA	R:55.25							
SCF91821	(TG)9	F:TTCTGTGTCTGATTCCATCTC	F:55.16	302–304	KP278792	2	1.55	0.31	0.36	0.29
		R:ACTAGCCCAACAACTTAGACTG	R:55.42							
SCF92414	(AG)12	F:GTTATCCTCCCTTTGATATGTG	F:55.29	283–287	KP278793	3	2.15	0.54	0.54	0.47
		R:AAGAGCAACAAGATGGGTACT	R:55.57							
SCF92564	(CTT)9	F:TCATAACTCCCTCGTAATCAAG	F:56.7	181–194	KP278794	5	3.89	0.92	0.74	0.70
		R:AGGAAGAAGAGAATAAGGTTGG	R:55.85							
SCF94237	(TTA)14	F:ATCGCATCAGGTAAGCTAGTAT	F:55.0	330–363	KP278795	8	5.20	0.92	0.81	0.79
		R:TCGAGTGTCATTGTAATAGGC	R:55.42							
SCF95754	(TC)12	F:CAGTGAGACTTCAGCTTGATAC	F:54.34	339–366	KP278797	6	3.49	0.85	0.71	0.68
		R:ATTGGTGACTTAGGAGTGAGAC	R:54.97							
SCF95767	(TA)9	F:TGAGGAGAGGAGTATCCATAAG	F:55.17	270–302	KP278796	5	4.12	0.62	0.76	0.72
		R:CCTACAAGTCTCGCAATTCTA	R:54.91							
SCF95851	(TG)9	F:GACCTTGGAATTTGATGATG	F:55.38	188–190	KP278798	2	2.00	1.00	0.50	0.38
		R:TGTAGATGGATGTTGTTACCTG	R:55.20							
SCF96311	(TC)9	F:TGTATAATCTCAGGGGCATT	F:54.68	155–161	KP278799	4	2.18	0.62	0.54	0.79
		R:TTTCTCATTTCCTTCCCAC	R:55.6							

Table S2. *Cont.*

Primer ID	Motif	Primer Sequences (5′-3′)	TM (°C)	Allele Range	GenBank Accession Number	Heterozygosity				
						N_A	N_E	H_O	H_E	PIC
SCF965	(GA)14	F:GTAAACTAACAAGCAACGATCC	F:55.73	258–275	KP278594	6	5.37	0.85	0.81	0.79
		R:GATTTAGCTGATGCAGAGTCAT	R:56.22							
SCF96539	(TG)9	F:GTAGCATAACCACCTCTTATCC	F:54.67	259–261	KP278800	2	1.08	0.08	0.07	0.07
		R:ATCTTGATGACTGTGTAAGCTG	R:54.64							
SCF9709	(AT)9	F:CCATTAGAAGAGTTTACCGTGT	F:55.20	270–272	KP278627	2	1.84	0.10	0.46	0.35
		R:TTATCAGTCCCTTACTCAATCC	R:55.2							
SCF97378	(CA)14	F:GTAGAGATCGTTGTCGTCATTT	F:55.98	233–248	KP278801	6	2.77	0.69	0.64	0.60
		R:AACATCGTGGTGTATTGGAT	R:55.19							
SCF9815	(GA)11	F:CATAGGAAGATTGCCTTGAG	F:55.5	186–194	KP278628	2	1.99	0.62	0.50	0.37
		R:GCCTGTTCACATAGATGGAG	R:55.67							
SCF98180	(GA)10	F:CTCCTCTGCTTATCTCTTCAAC	F:55.11	345–347	KP278802	2	1.45	0.39	0.31	0.26
		R:GGTTTTCCCTTCTCAAGATTAC	R:56.7							
SCF98686	(TC)11	F:CGTAATTTCACATCCTCGTT	F:55.25	236–253	KP278803	4	3.60	0.92	0.72	0.67
		R:CATAACCAGATAGCACCTCAAT	R:55.94							
SCF9872	(TC)11	F:ATGGGAGTGCATGAATAAAC	F:54.99	234–238	KP278629	2	1.26	0.23	0.20	0.18
		R:GGAGAATCGTATTTGTGAAGAG	R:55.63							
SCF9909	(AG)11	F:CGTAGGTGGATTTCTCTACAAT	F:55.13	134–173	KP278630	6	5.45	0.85	0.82	0.79
		R:GGCATCTTATTTATCGTCTCTG	R:55.74							

Note: * = marker displayed segregation distortion $p < 0.05$.

Table S3. Primer characteristics for primer loci tested and validated on 4 craunberry cultivars, but not used in genetic diversity or allele segregation analyses.

SSR ID	Motif	Sequence	TM (°C)	GenBank ID
119364_K70	(CT)18	F:ACCACAAAACCCTAGTTCTATC	F:54.17	KP279272
		R:TCCATAGTCTTAGCAACAACAG	R:54.84	
1trimcontig175833	(TG)14	F:CTCTTTCTGCCTGGTTCTAA	F:54.84	KP279278
		R:ACTACTATTGCGTATGGCTCTT	R:55.08	
1trimcontig178732	(TC)15	F:ATGGTCCCTGAGTCTAACTTC	F:54.88	KP279279
		R:GGATCTCTATTTCAGTGTGTTG	R:53.96	
1trimcontig217288	(AG)17	F:ATAACAGAGGACAACGATCTG	F:54.37	KP279280
		R:TCACTCTACTTTTACCGAGACA	R:54.39	
1trimcontig240704	(GA)16	F:GAGAGAGGGAAGAGTAACAGG	F:54.76	KP279281
		R:AAGATGGTCTATTGAGTATGGC	R:55.04	
1trimcontig241039	(GAA)11	F:ATAATGGACTGCACGAAACT	F:54.81	KP279282
		R:GTAGTAGGGATTTCACAGGCTA	R:55.28	
1trimcontig336911	(TC)14	F:CATTTCCTATTTCATCCCCT	F:55.58	KP279283
		R:AACAGAGCGAGAGTAATTGAAG	R:55.62	
1trimcontig354570	(TG)14	F:ACCTGTTCTGTTGATTACGAGT	F:55.50	KP279284
		R:ACAGTATCGCACAATGAGTTC	R:55.32	
1trimcontig439506	(AG)18	F:GATTTAGGTTAGGGTATGGGT	F:53.92	KP279285
		R:GCTTGTGTTAGGGTTTGTTA	R:53.03	
1trimcontig445838	(CT)14	F:GTTTTCCTCTGAATCTCCACTA	F:55.20	KP279286
		R:GTCATACACAATACACAGTCGC	R:55.71	
1trimcontig448145	(AC)15	F:TGTGATTAGAGGGAGGATTTC	F:55.80	KP279287
		R:AAATAAGGGAGTTTGAACCG	R:55.88	
204816_K70	(AG)14	F:CACTCTAATCACCCTTTCACTC	F:55.55	KP279273
		R:CAGAGAGGAATAATACAGGTGC	R:55.67	
239628_K63	(AG)16	F:CTCTTTCTTGGATGTTGCTACT	F:55.47	KP279257
		R:CGAAACTCTCTAACTCTGGTGT	R:55.38	
247873_K63	(TC)15	F:GATCGGAGAGTTTTCCTCTT	F:55.07	KP279258
		R:CAATTTCCTTCCCCAACTAT	R:55.66	
24956_K70	(GA)14	F:AGAGAGAGGATTGTTATTGCTG	F:55.39	KP279270
		R:TGAACCAAGCCCATATAAGT	R:54.77	
281741_K70	(GA)17	F:GATTTGACTCGTAAAGCAGAC	F:54.26	KP279274
		R:GGAAATGGAGATGGATATGTAG	R:55.21	
284499_K63	(AG)15	F:ATTAGTTCTCCTATGTGGCTTG	F:55.28	KP279259
		R:TCAGAGCTTACCCTATTTCAGT	R:55.01	
289194_K63	(TC)14	F:CTAGCACTGGCTCTTACCAC	F:55.22	KP279260
		R:TGTAGGATGTGTATATGGAGCA	R:55.73	
307461_K70	(TC)17	F:CAGACACTCCACTAACTCAGAA	F:54.67	KP279275
		R:GCATCAACAGTACAACAATACC	R:54.78	
307534_K70	(AG)15	F:ATCGTCTGCTATAAATACTCCG	F:55.39	KP279276
		R:GTGTCAACCTTCCTTACAAGAT	R:54.99	
310238_K70	(AG)17	F:GAGTAACAACAGTGGCAAAAC	F:54.96	KP279277
		R:AACTTCCTCATGTACTTTCCC	R:54.90	

Table S3. *Cont.*

SSR ID	Motif	Sequence	TM (°C)	GenBank ID
339139_K63	(CT)18	F:CTAATACTTTCATCGTCAACCC	F:55.52	KP279261
		R:AGGAGAGAGAGAGGTAGTTTGG	R:55.93	
346445_K63	(GA)16	F:TAAGGGAAACCTGTAAAGACG	F:56.12	KP279262
		R:GATAGCAAAGTGGACGAGTATT	R:55.27	
35137_K63	(CT)14	F:GGAACATCAAAACTCCCATAC	F:55.99	KP279254
		R:GTTCTTCCCCATTTCAGTAAGT	R:56.41	
36394_K70	(AG)18	F:CAGTGTTTGTTGCTTGGTC	F:55.07	KP279271
		R:ATCTCACTCTCTGTTTCCCTC	R:54.98	
37487_K63	(GA)15	F:CTTTCATTAGAGGAGAGCTTGT	F:54.74	KP279255
		R:AGGAAACTAGCAATCAGTCAAC	R:55.30	
389746_K63	(CT)17	F:TTGTAAACCTCAAGACACACC	F:55.21	KP279263
		R:TATCACACAGTTTTGGAGAGAG	R:54.62	
413893_K63	(TC)21	F:TACTCCATTTCACAACACGA	F:55.02	KP279264
		R:ATCTCTGCTTCTTCTACCTCTG	R:54.71	
418138_K63	(TC)16	F:CCTCTTCTTCATATCATCCAGT	F:55.00	KP279265
		R:TTTAGCCCACTTTTATGCAC	R:55.57	
418192_K63	(GA)18	F:CAGGCAGAAGAAGAAAGAAA	F:54.97	KP279266
		R:TGAATTAAGAGAGGAGGAGAGA	R:55.01	
418730_K63	(CT)17	F:ACAGATCCAGTCTCTTCAAATC	F:55.02	KP279267
		R:ATACGGAGTGTAGATGTCTCCT	R:54.53	
419957_K63	(GA)14	F:AGACTCACCTCTCTTTCTTGTG	F:55.34	KP279268
		R:GACTATCTTTCGGTTGACACTT	R:55.15	
49132_K63	(TC)18	F:AACCCTAGAAATCAATGCAC	F:54.29	KP279256
		R:GTTTTCCGTTTTGTTCTGTC	R:54.82	
9053_K70	(AG)15	F:GCTGATTAGGTTCACTTTCTTC	F:54.96	KP279269
		R:TTTCTTCACCTCTTTCTCTCTC	R:54.64	
ct117109	(TC)9	F:TTGacGTCTTCTCTCTCTTTCT	F:55.14	KP279151
		R:CTAGGGTTCATACTTCGAAAAG	R:55.04	
ct118488	(TC)9	F:GTTCAGGACAAGTGATTTtCTC	F:55.51	KP279152
		R:AGCTAAGTGtTTTcCtACTGGa	R:55.09	
ct131127	(AAT)6	F:GAGTAGTCCcGtAtAtGGAaTg	F:55.32	KP279153
		R:GTTCaTTTCCCCATTCTGA	R:55.81	
ct136900	(AAAAGG)4	F:ACGATATGAGAGAAGAAGAGGA	F:54.87	KP279154
		R:CTCTAGTGCATACCAGCACTT	R:54.83	
ct139597	(GA)11	F:CTTATAGGCAATGCACATAcAC	F:55.08	KP279155
		R:GTAACTAATGGGGCTGAACTT	R:55.11	
ct142970	(ATATA)4	F:AAACCTAAATaCCCGGAATG	F:55.69	KP279156
		R:TATAGAcgGCAtATGCAACA	R:55.29	
ct146598	(AC)13	F:AATCTCATTTTTCCTGggTC	F:55.62	KP279157
		R:CTTGATATgCtcTCTTAATGGC	R:55.89	
ct170930	(AAG)7	F:ACCcGATTCCATAAAAGAAG	F:55.81	KP279158
		R:CAAGCTTCTCCTaCCTcca	R:55.53	
ct89569	(AC)9	F:ACTAATCCCacgAAAACTGA	F:54.79	KP279149
		R:aTCTaggCtTTCAAACtAGGGT	R:55.49	

Table S3. *Cont.*

SSR ID	Motif	Sequence	TM (°C)	GenBank ID
ct97791	(AC)10	F:GACTTTGTGAGGATAGACCATT	F:54.91	KP279150
		R:ATGTAAGATGTGGaCaTaAgGG	R:55.63	
SCF101363	(TC)9	F:CGATCTGTATCTAGTCGTGATT	F:53.70	KP279053
		R:GAGATGTACTATTGGAACTTGG	R:53.15	
SCF102190	(GA)11	F:GAGGAAAGGGTGAGAGTTTT	F:54.99	KP279054
		R:GTTTGACGAAAAGGAGACTG	R:54.98	
SCF102509	(CT)10	F:ATAGGATTTGTTAGACTTGGGG	F:56.36	KP279055
		R:GGAGCTGTTGAAGCTATTGTTA	R:56.42	
SCF102538	(GA)12	F:TTACTGGGCAATAGAAGGACT	F:55.66	KP279056
		R:CACATAAGTTTGGCTACACAAC	R:55.03	
SCF108101	(TC)14	F:AAATCTTCCATGAGCTTGTC	F:54.38	KP279057
		R:TACTGCGGTGTTGAATTAGA	R:54.44	
SCF113304	(ATA)24	F:CCAGTCAACGAACAAATAGAG	F:55.06	KP279058
		R:CCTAAAGGGAAAGAGAAGTGA	R:55.36	
SCF113895	(AG)10	F:GGAATCACTATGAACATGCAC	F:55.50	KP279059
		R:ATCAGAAACGAGTCCAAAGAC	R:55.95	
SCF115821	(CT)10	F:TCACCACTTACAACATATCCAC	F:55.03	KP279060
		R:TTGACACTAGCAAATTCCATC	R:55.39	
SCF117	(CA)12	F:ATAGCATCTGTCTTATTGGACG	F:56.09	KP278954
		R:GTGGGTTTCTGATCTTCATCT	R:55.71	
SCF118209	(GA)15	F:AGGATTTAGGACGTTGGAA	F:54.69	KP279061
		R:GTAACAGAGAAAGCGAGAGC	R:54.55	
SCF118536	(GA)10	F:GGGTACTATATGAAGGTGCCTA	F:55.10	KP279062
		R:CTACCATGTAACCCTTGAAAGT	R:55.05	
SCF118603	(TCT)13	F:GGACAAACACTAAATAAGCCAC	F:55.58	KP279063
		R:CTGCTCACAGAATACCACTAAA	R:54.84	
SCF119813	(TG)10	F:GTTAGTCGGCTCAAGTTAGTTC	F:55.07	KP279064
		R:ATGGACTTCCCATTTCTTTC	R:55.62	
SCF119984	(GA)10	F:TTAGAATTGCGTTCCATACAG	F:55.59	KP279065
		R:GAAAATCAGTTCGATTCAGGT	R:55.86	
SCF12084	(TC)12	F:CTCTTGTTGGACGGATCTATT	F:55.90	KP278974
		R:CCTAACATTTCTCCCACTCA	R:55.21	
SCF122440	(GA)12	F:CTAATCTTCCTCCTCTTGTTGA	F:55.37	KP279066
		R:CGACAAACTAACATATCATCTCC	R:55.53	
SCF122552	(AC)10	F:TATATCGAGGTCATTGCGA	F:55.15	KP279067
		R:GAGTTGTCGTTAAGGTTTTGA	R:54.62	
SCF123643	(GA)9	F:CAAGAATGAAGAGAAAGATTCC	F:54.86	KP279068
		R:CAGGTTTTATTAGCCTGTGTTT	R:55.36	
SCF125768	(AG)11	F:CTCACTTCTCATACAACATTGG	F:54.91	KP279069
		R:CACAACAGAACCATCAGTACAT	R:55.09	
SCF126993	(AC)11	F:TTATGGCTCTCATTAAGCAAG	F:55.00	KP279070
		R:CTTATTTGGGGTTGATGTGTA	R:55.12	
SCF127382	(TC)10	F:GTCTTTAGTGCTGGGTTAAAAG	F:55.10	KP279071
		R:TGATTTCTAGTGTCTCCTCTCA	R:54.23	

Table S3. *Cont.*

SSR ID	Motif	Sequence	TM (°C)	GenBank ID
SCF12818	(CT)9	F:GTGAGGGAGAGTGTTAGATAGC	F:54.82	KP278975
		R:ACAAGAGAAAGAACGACAAGAC	R:55.34	
SCF128658	(AG)9	F:ATTATTGATGAGTAGTCCCCAC	F:54.73	KP279072
		R:TGGTTGATTTGTGTAGAAAGAA	R:55.06	
SCF13045	(CA)9	F:TGTCCAGTGCTAATATCTGTGT	F:54.93	KP278976
		R:TCCTCCAAATCTATGCAAAC	R:55.29	
SCF130555	(ATT)8	F:GGGTAAAATAAAAGGTTCTCC	F:53.82	KP279073
		R:TCCTTACTTGTCGATTAGGC	R:54.64	
SCF130642	(GA)9	F:AGGCGGAAGATGAAAGTAAT	F:55.56	KP279074
		R:TGTCAACATAAAACGATAGCAG	R:55.68	
SCF132006	(GT)9	F:ATTGAGGTCACTAGGAGGTGTA	F:55.43	KP279075
		R:GAGGAGAGTGTTTATGTTCATGT	R:55.00	
SCF13231	(CT)12	F:GAAACAAAGAGGAGAAGACAAC	F:54.83	KP278977
		R:GTGAAAGGTAAGAGATGGGTAG	R:54.70	
SCF132852	(TC)13	F:TGCTTGTGTTAGGGTTTGTTA	F:55.99	KP279076
		R:GTTAGAGATGATGGCTGAAGAT	R:55.15	
SCF132868	(TG)9	F:CTGATTTGTGTTGATGGATAAG	F:54.85	KP279077
		R:CAGTTAGCACCACCTAGTTAGA	R:54.06	
SCF133587	(AG)10	F:TTAAGCACCAACACTAAATCC	F:54.60	KP279078
		R:AGTTCATGTGACGTTGTATCA	R:53.96	
SCF13393	(AG)16	F:ATATACACAATCGCACGAGAC	F:55.28	KP278978
		R:TCAGCTTACGATCTCACAAA	R:54.54	
SCF134365	(CTT)8	F:GCCTTGTTATGTTACCTGTGA	F:55.34	KP279079
		R:ACAACTATCTGGAAAAGGGTT	R:54.52	
SCF134906	(ATA)7	F:GTATGATTGGTCTTGGTCTGAT	F:55.59	KP279080
		R:GCAACAGCTAGAGATGCTTAAC	R:56.18	
SCF13628	(CT)12	F:AGAGGTCAATAGCTGAAGAAGA	F:55.11	KP278979
		R:AATTCCTGTAGTAAACAGTGGG	R:55.05	
SCF13665	(GT)9	F:TTCTTTACTATACCCACAACCC	F:54.99	KP278980
		R:GTTTCCTAAGAGCATCAACAAC	R:55.70	
SCF136684	(CT)12	F:TCTTATCCTGCTTTCTTACCC	F:55.31	KP279081
		R:ACAGGGTCATTACTGTCTTGTT	R:55.34	
SCF14090	(AC)13	F:GTATTGTCTGGAGATTCCCTAA	F:55.02	KP278981
		R:GCTCTTTGCATCATACTCAA	R:54.02	
SCF142636	(AC)10	F:GGTCATGGTGTCATTCAAG	F:54.50	KP279082
		R:CATGGACAGGTATTGGACA	R:55.04	
SCF142785	(AG)9	F:AGGCTCACATTTCTAACTCAAG	F:55.47	KP279083
		R:ATATCTACCTCCCTAATTTCCG	R:55.62	
SCF14358	(AG)10	F:CCACTAAAACCCTATACTTGGA	F:55.15	KP278982
		R:GTCACTTTTCTATTGCTGGTG	R:55.05	
SCF145195	(GA)14	F:CCACCTTCCATTATACAGCA	F:56.13	KP279084
		R:GAACAAGAGAAGAACCCAGATA	R:55.20	
SCF14690	(CT)9	F:CCTTCCATCTTCTTCTTCAAC	F:55.59	KP278983
		R:AACAAGGTTAGGAAACTAGGGT	R:55.28	

Table S3. *Cont.*

SSR ID	Motif	Sequence	TM (°C)	GenBank ID
SCF147678	(CT)11	F:ATTCATAGTTTACCCGTCACTC	F:54.96	KP279085
		R:GATTGCTGCTCTTTCAATGT	R:55.52	
SCF14838	(GA)9	F:ATAATTTTGTCCCACACGG	F:55.78	KP278984
		R:TGAGAGTTCAAGGGCAATAA	R:55.97	
SCF14877	(TG)10	F:CCCATGATCCTATGTATGCT	F:54.95	KP278985
		R:AGCTCTGATACCAAACTGTCAT	R:55.58	
SCF148938	(AT)10	F:CTTCTGTCATTTTAGTGTCCTG	F:54.23	KP279086
		R:ACCTTTTGAACACATTGGAC	R:54.93	
SCF149145	(CT)12	F:CTTCAACATATACCCACCCTAT	F:54.80	KP279087
		R:GACCAAAACTAGAAAACTCCCT	R:55.76	
SCF150395	(TC)12	F:CTCTGGTTCATCCCTCTGT	F:54.97	KP279088
		R:CAGACCCTGTCGTTACAAAT	R:55.15	
SCF150410	(TAC)7	F:AACGTAGACACGAAAAGAAGAC	F:55.38	KP279089
		R:GCTAGACATGGTTGGAAGAC	R:54.75	
SCF15112	(GT)12	F:GTATTGTGAGAGGATGACCTG	F:54.60	KP278986
		R:AAGGGCTTTAGTGTTGTTGT	R:54.05	
SCF15143	(AT)10	F:AAAGCCTGCAAATACTCCTA	F:54.42	KP278987
		R:CAAACTAGGTCACAAGCACTTA	R:54.92	
SCF152348	(TC)9	F:AGGAGCAAGAAGAGGTGTTT	F:55.68	KP279090
		R:CCATTGTTTTGCACTTCAG	R:55.18	
SCF1524	(GA)12	F:TAACATACAGTCCTCGACAAGA	F:55.07	KP278957
		R:GATCTAGTTGTTCTTCCGCAT	R:56.05	
SCF15729	(GA)13	F:GAACTGGCTCACTAAAAGAACT	F:54.65	KP278988
		R:GGTGCATAGCGATCTTACTATT	R:55.38	
SCF157301	(AG)10	F:CGTTACATACTCCACCCAAT	F:54.95	KP279091
		R:GATTTCAAGAAGGGTTTGTG	R:54.75	
SCF157676	(TC)10	F:GTCCGCAGTGTCTATGTTT	F:53.53	KP279092
		R:CATACCTTAGATGGTGATTAGG	R:53.18	
SCF16186	(CT)10	F:CTTGTATCAACTTCCATCGTCT	F:55.99	KP278989
		R:CCAAAACCTGAGAAACTTAGAG	R:55.06	
SCF164500	(AG)10	F:AAATCACCATTCTGGAACAC	F:54.84	KP279093
		R:GGTCGGAATACTAAAACAGAGA	R:55.25	
SCF165	(AG)9	F:CCTCCTCAATCTTCTTCTCC	F:55.50	KP278955
		R:TATCTTAAACGGCTGATCTCTG	R:56.36	
SCF171768	(AG)13	F:GTATCCCCTTATACAACCTGC	F:55.30	KP279094
		R:GGCTTCTATTATTCTATTGCCC	R:56.25	
SCF18113	(AG)11	F:GAAGACATCAAAACTGGGACT	F:55.79	KP278990
		R:TCAGATCAACACTGGACTAAGA	R:55.02	
SCF186078	(GAA)10	F:TAGAAAGCAGTAGAGGAGGAAA	F:55.29	KP279095
		R:CTTTTCGGATCTGTTTGGT	R:55.19	
SCF19055	(CA)10	F:AACTCTCACCAAAGGTATTGTC	F:54.99	KP278991
		R:GTGACGCTAGATGAGGACTTA	R:54.71	
SCF195276	(GA)9	F:GTACACTCAAAAGGGAAGAAAC	F:54.74	KP279096
		R:TGGCGTATGAGAAGAAGATT	R:55.04	

Table S3. *Cont.*

SSR ID	Motif	Sequence	TM (°C)	GenBank ID
SCF197012	(CT)9	F:GTACGATACAACATGGACACA	F:54.28	KP279097
		R:ATATAAACAGGGATGCGACT	R:53.86	
SCF19788	(TC)13	F:TTCTCTGACTTGTCTCGACC	F:55.40	KP278992
		R:CATTCCTGAAAACAACTACTCC	R:55.56	
SCF203038	(TC)12	F:CACTTCTGTACCCTCTTTTACC	F:54.78	KP279098
		R:GTCTCATACCTGAATTTTCTGC	R:55.62	
SCF221037	(TC)9	F:ACACTACAAGCAACAGACAAGT	F:54.21	KP279099
		R:TAGTCGAGGTGTGCGTAAG	R:54.95	
SCF22339	(AG)11	F:CCTCAATCTTATGGATCGAA	F:55.22	KP278993
		R:TTGTAGAAGAACCTGTAATGGG	R:55.99	
SCF2270	(AG)12	F:TTGGTGTAAAGAAGGATAGGAG	F:55.26	KP278958
		R:GGCTCCAACTAATGCTATGA	R:55.49	
SCF22993	(CTT)7	F:GACTGTGCGTAGACTTGATCT	F:54.58	KP278994
		R:AAGTATGTGTAGGCCGAAAA	R:55.01	
SCF24570	(TC)11	F:GGATGCTGTCAAAGATATTG	F:53.18	KP278995
		R:TAAACAACTGAGAACTGTAGGC	R:54.01	
SCF27509	(AG)12	F:ACCAGAAGAACCATGAACTG	F:55.10	KP278996
		R:CAAGAAGCCTGATATGTTGTC	R:54.97	
SCF2942	(GA)11	F:ATAAGATCGGTGAAGGATAGG	F:54.96	KP278959
		R:AAGGAGATTAAGAAGGTCCAAG	R:55.85	
SCF29521	(AG)10	F:CTCAATGCTTCGGAGTAGATA	F:54.82	KP278998
		R:CTCCTTGTTTTCACAGGTATG	R:54.90	
SCF29529	(AG)12	F:AACAGGGAGTTTTCCTACTCTT	F:55.38	KP278997
		R:GTATGATGGGAATGGGATAGT	R:55.30	
SCF30167	(TC)11	F:AGACATACGAAGTCCATGAAAC	F:55.82	KP278999
		R:CACCCATAACTCACCTCTAATC	R:55.36	
SCF30716	(TC)9	F:ATCGGTGACAAAGGTAGATACA	F:56.26	KP279000
		R:TGTCTAGGTTGAAAACAAGGAG	R:56.19	
SCF30747	(AG)10	F:AAGTCAACCAATAGGCATAGAC	F:55.12	KP279001
		R:TGTAGTAGCAAGCAAGCTGAT	R:55.57	
SCF3187	(GA)11	F:CCAGAAAACTACAGATACCCTC	F:54.70	KP278960
		R:GTACTTACCGGGACAACTCTTA	R:55.20	
SCF32389	(AG)10	F:CACTATATCCTACCCCAAAGAG	F:54.69	KP279002
		R:TTTGCTGTGACTTGAAAGG	R:54.87	
SCF32769	(AG)13	F:CTTACTGCCTTACATCCTCTTT	F:54.64	KP279003
		R:CTGGCAAATAGCTTACAGAAC	R:54.52	
SCF33205	(TC)9	F:ATTCTGACTGTTTCATTGCC	F:55.19	KP279004
		R:AAATGTATTGGTGGGGAAGT	R:55.85	
SCF33518	(AG)9	F:GTATTCGTACTCCACACCCTT	F:55.69	KP279005
		R:TACAGACAACCATACATTAGCG	R:55.56	
SCF33654	(CT)11	F:CACAGCCTTAACACAGGATT	F:55.32	KP279006
		R:GTGGCTCCTTATCTGGGTA	R:55.14	
SCF34663	(CT)10	F:GTTTAAGTTCTAGCATAGCCGA	F:55.72	KP279007
		R:GTACACAAATACAGAGTGTGGC	R:54.84	

Table S3. *Cont.*

SSR ID	Motif	Sequence	TM (°C)	GenBank ID
SCF3507	(CA)9	F:GCTAATAAAGGTTGAAGTCTGG	F:55.03	KP278961
		R:CCATGTAGTAGTGAGAGCTGTG	R:55.18	
SCF35370	(TC)9	F:AATCATGGTCTTCTCACGTT	F:54.57	KP279008
		R:GTATAATTGCGTAAGTGCTCG	R:55.38	
SCF36355	(CT)9	F:GTGAAAGGACTGTTTTACCCTA	F:55.17	KP279009
		R:GAGGAGGGGTTTCTCTTTT	R:54.90	
SCF36716	(TG)9	F:CTAGGCAATGATGACAAAGC	F:55.99	KP279010
		R:CCCAATAGTTACCACTAAGCAT	R:55.17	
SCF36905	(AG)9	F:GATAAGCTGTGCTGAAACATC	F:55.12	KP279011
		R:CGATAGGGGATAGAATTAGTCA	R:55.15	
SCF39229	(TC)12	F:AAAAGCTACGATACGAATGC	F:54.85	KP279012
		R:AGAAGGAGATAGTCAACGAATG	R:55.24	
SCF39691	(AG)9	F:TAAACCATAGTCCTCTCCTCC	F:54.99	KP279013
		R:GTCCATAACTCCAAATAAGAGC	R:54.79	
SCF40225	(GT)11	F:GCTTGACTGGATTAGAACAACT	F:55.30	KP279014
		R:CTGGGTATCAACATCAACAGTA	R:55.20	
SCF41166	(TAA)7	F:TTCTAACTCAAGTAGTCCTCCTG	F:54.69	KP279015
		R:AGAAGAACAGCAGATTCCAC	R:54.44	
SCF41759	(GT)10	F:TTGCCATCTTCTTGTTCTTC	F:55.50	KP279016
		R:ACAACATGCTAATGTGGGTACT	R:56.20	
SCF42256	(GA)9	F:ATACTGCTCAACTGATTTAGGG	F:55.28	KP279017
		R:CGGAGGAAAGTCTGCTATATT	R:55.34	
SCF42549	(CT)10	F:CTCTTCAGCCCTAATCATATTC	F:54.87	KP279018
		R:CAGGACAAACATCTAGGTCAA	R:55.31	
SCF4283	(GAA)7	F:CTTGACTTTGTAGTGGTGTTTG	F:55.08	KP278962
		R:CTCCTCCCCTCTTTTAACTAAT	R:55.04	
SCF43996	(ATT)10	F:ATAGTGTATGTATCAGAGCGGG	F:55.97	KP279019
		R:TATTTACGGGAGGTGTGAACTA	R:56.40	
SCF47689	(CT)12	F:TGTAGATGCCGTCAAAGAAT	F:55.83	KP279020
		R:ACTATAACTCCAAGCGCAGTAT	R:55.08	
SCF48612	(TTC)7	F:GAACACGATTTGACATTTCC	F:55.45	KP279021
		R:CTCCTATGTTGTTTTCGTCTGT	R:56.06	
SCF50668	(AC)11	F:CTTAATATGTCCTAGCCCAAAC	F:54.86	KP279022
		R:GAGGTGTAGAATAGTGAAAGTGG	R:54.79	
SCF51607	(AG)10	F:TTATGTAACTGACGCTGATAGG	F:54.82	KP279023
		R:GTAGATTTGCGATGGTGTATG	R:55.71	
SCF5230	(CT)11	F:TTCAAGATGCCTAAACCAGT	F:54.95	KP278963
		R:GTATAGTGGAGAAGAAGGGTGA	R:55.08	
SCF53058	(TC)11	F:TACCACAGTCTCCTTAACAAAG	F:53.86	KP279024
		R:CATACTCTATAATCCACTTCCG	R:53.34	
SCF54555	(CT)10	F:TTACCAAAGCACCCATTAAC	F:55.26	KP279025
		R:ACGACACATATCTCCAAAGTG	R:55.16	
SCF55619	(CT)9	F:CAAAGAATCAGCAGGAGGT	F:55.35	KP279026
		R:GATGTCTAAGGTACAAGGAAGC	R:54.84	

Table S3. *Cont.*

SSR ID	Motif	Sequence	TM (°C)	GenBank ID
SCF5899	(GAA)8	F:TAGCCTTGGGTATTAGAACACT	F:54.91	KP278964
		R:GGAAGACAAGACAAGAGGC	R:54.84	
SCF5935	(GA)9	F:CTGAACTGAAACACCAAGAAC	F:54.91	KP278965
		R:AGAAATGAGACCTACACTGCAT	R:55.58	
SCF6050	(TC)9	F:TAACAAAATAGAGACCTCCCTG	F:55.26	KP278966
		R:TTGACTGGTTGATGGTGTATAG	R:55.20	
SCF6053	(CT)13	F:GTTGAAGCATCCTACTCAAAAC	F:55.70	KP278967
		R:CCTAGTGAACAGTCATTTCCTT	R:55.16	
SCF61946	(TG)9	F:GGATAAAAGGGTACTCCATACA	F:54.92	KP279028
		R:GGTTCATAGTGGCGAAATTA	R:55.34	
SCF61972	(GA)9	F:CAGATGAATTTAGACGAGTGG	F:54.98	KP279027
		R:TGCATAGCTCAAATATCCCT	R:55.01	
SCF63953	(GA)9	F:GTTGGTGTGGTTCTCATTATC	F:54.45	KP279029
		R:GTGAGTGCTGATAGGGTAGAGT	R:55.17	
SCF65004	(TTC)9	F:GAATCAATCCAGTCCATAGG	F:54.44	KP279030
		R:CTTACACCACTCTTCCCAAC	R:54.68	
SCF65897	(CT)10	F:CTTATTTTGCTGAACCTTGG	F:55.15	KP279031
		R:CTCTAACTATTCTTGCAGCCTC	R:55.31	
SCF662	(GA)10	F:TTGGACAATCTTACCCATAGAC	F:55.76	KP278956
		R:CTTGGCGTGCATAGAATAA	R:54.99	
SCF66313	(CT)10	F:AATTTGACCCTCTTTCCCT	F:55.19	KP279032
		R:GTCCAAAATACACAAACTAGCC	R:55.58	
SCF68007	(GAG)7	F:CACCCACCACAACATAAAC	F:54.49	KP279033
		R:TCTTCTACTGAACAGCTTCTTG	R:54.27	
SCF6819	(CA)9	F:TCATCATCACTCCAACTACAGA	F:55.75	KP278968
		R:TGTAAATTCTGAGCCCTTGT	R:54.95	
SCF74917	(TA)11	F:AAACATAAAGAGCAGCCAGTAG	F:55.65	KP279034
		R:CTGATAAATAGAGACAGACGGG	R:55.68	
SCF76055	(GT)9	F:GATCGAAATGAGGATTTGTG	F:55.55	KP279035
		R:CTCTTCCACTGTCAACTTTTCT	R:55.35	
SCF77055	(CT)9	F:GAACTGGTAAGGTTTGGAACTA	F:55.17	KP279036
		R:CTTGGAAAGGATTCATACTAGC	R:54.95	
SCF77382	(CA)9	F:GTTTTCCACAAATCTAGTCGTC	F:55.55	KP279037
		R:TGTGAGACCAAAGTGACAAG	R:54.58	
SCF7822	(GA)9	F:GTCACTCATGGTAGTATTGTACG	F:54.02	KP278969
		R:AGTCTTACGTTTGGTGTCG	R:53.66	
SCF80777	(GA)10	F:GAGGCAATGTTAGTCTTTGGT	F:55.97	KP279038
		R:GGATACAACAGCTAGAACCACT	R:55.18	
SCF8189	(GA)10	F:GACAAGGAGGAAGAAATAGTGA	F:55.20	KP278970
		R:ACCAGCAGAAGCAGTTAAAG	R:54.95	
SCF83079	(AC)12	F:GTATTCACCAAATCTACCCAGA	F:55.76	KP279039
		R:GTTAAGGATTGTGTCCCTCA	R:55.03	
SCF83872	(TC)10	F:GGAGCTTGAAAACCTAAACA	F:54.69	KP279040
		R:GTTAGTGAGGAGGGGAGAG	R:53.71	

Table S3. *Cont.*

SSR ID	Motif	Sequence	TM (°C)	GenBank ID
SCF84796	(TC)10	F:CTACTCTTAGGGCATCTCCA	F:54.62	KP279041
		R:CAACACTACTGACCTTCACAAT	R:54.39	
SCF84921	(TCT)7	F:CTTCATCGTCTTATCAGGTTG	F:54.98	KP279042
		R:CCAAGTGAGTCTGTGATTGAT	R:55.11	
SCF85469	(AC)15	F:CCAGATAAGTAACACAACACCA	F:55.28	KP279043
		R:GGGAGTGCTCATTTGTAGTC	R:54.75	
SCF85776	(AG)9	F:CTAAGTTCCAAACAGAGCCTTA	F:55.63	KP279044
		R:AAGTTACCACCGCTAAGAAAC	R:55.33	
SCF87305	(CT)11	F:AATGCTCTCCAGACTTTTCTAC	F:54.57	KP279045
		R:GTGCAGTATCAAATGTAAGACG	R:55.11	
SCF87786	(CT)11	F:AGGGAGATAGTTGTTCCCAT	F:55.07	KP279046
		R:GCCTAAACCTAGTAAACTCTGC	R:54.43	
SCF9045	(GA)10	F:GCAAATGTCACTGTTAGGATAC	F:54.03	KP278971
		R:GAAAGGAAGAGAAGTTAAGCAG	R:54.49	
SCF9100	(TAT)15	F:TTAGTCCCACTCCTCAATTATC	F:55.02	KP278972
		R:GGGCTCACTATCACTACTCATT	R:55.10	
SCF91560	(AAT)10	F:TATTAAACTCACTGCACCTCTG	F:54.84	KP279047
		R:TGACCATCTATGAGAAGCTATG	R:54.67	
SCF92986	(AC)9	F:AACTAACCCGGACACCTAGTAT	F:55.94	KP279048
		R:CGAGGGAGACAATATCAAAGTA	R:56.07	
SCF9350	(TG)9	F:GGATTTACCACACCATTCTG	F:55.33	KP278973
		R:AAGAAATTACCACATGCACC	R:55.07	
SCF95879	(AT)9	F:TTTTACATGAAGTGGTAGAGGG	F:55.99	KP279049
		R:CCAGTTGTATAGATTTTGCTGG	R:56.54	
SCF96306	(TC)9	F:CCTGTAGTGAGTTACCTTCCAT	F:55.04	KP279050
		R:GCTGTCAACCATCCATTATT	R:54.99	
SCF99113	(GA)9	F:CATGACTTGCTTGTATGGTG	F:55.06	KP279051
		R:CACAACTCGCATAACTCTACTC	R:54.42	
SCF99997	(GA)9	F:ATAGGTCATCTCCTTCTTGTTG	F:55.08	KP279052
		R:ACTACTACCGTTGATTGCCTT	R:55.60	
